# Treatment with direct-acting antivirals improves peripheral insulin sensitivity in non-diabetic, lean chronic hepatitis C patients

**DOI:** 10.1371/journal.pone.0217751

**Published:** 2019-06-06

**Authors:** Giacomo Gastaldi, Diana Gomes, Philippe Schneiter, Xavier Montet, Luc Tappy, Sophie Clément, Francesco Negro

**Affiliations:** 1 Division of Endocrinology, diabetology, hypertension and nutrition, Geneva University Hospitals, Geneva, Switzerland; 2 Department of Pathology and immunology, University of Geneva, Geneva, Switzerland; 3 Department of Physiology, Faculty of Biology and Medicine, University of Lausanne, Lausanne, Switzerland; 4 Division of Radiology, Geneva University Hospitals, Geneva, Switzerland; 5 Division of Clinical Pathology, Geneva University Hospitals, Geneva, Switzerland; 6 Division of Gastroenterology and hepatology, Geneva University Hospitals, Geneva, Switzerland; Kaohsiung Medical University, TAIWAN

## Abstract

**Background and aims:**

Hepatitis C virus (HCV) infection is associated with insulin resistance, which may lead to type 2 diabetes and its complications. Although HCV infects mainly hepatocytes, it may impair insulin sensitivity at the level of uninfected extrahepatic tissues (muscles and adipose tissue). The aim of this study was to assess whether an interferon-free, antiviral therapy may improve HCV-associated hepatic *vs*. peripheral insulin sensitivity.

**Methods:**

In a single-arm exploratory trial, 17 non-diabetic, lean chronic hepatitis C patients without significant fibrosis were enrolled, and 12 completed the study. Patients were treated with a combination of sofosbuvir/ledipasvir and ribavirin for 12 weeks, and were submitted to a 2-step euglycemic hyperinsulinemic clamp with tracers, together with indirect calorimetry measurement, to measure insulin sensitivity before and after 6 weeks of antivirals. A panel of 27 metabolically active cytokines was analyzed at baseline and after therapy-induced viral suppression.

**Results:**

Clamp analysis performed in 12 patients who achieved complete viral suppression after 6 weeks of therapy showed a significant improvement of the peripheral insulin sensitivity (13.1% [4.6–36.7], p = 0.003), whereas no difference was observed neither in the endogenous glucose production, in lipolysis suppression nor in substrate oxidation. A distinct subset of hepatokines, potentially involved in liver-to-periphery crosstalk, was modified by the antiviral therapy.

**Conclusion:**

Pharmacological inhibition of HCV improves peripheral (but not hepatic) insulin sensitivity in non-diabetic, lean individuals with chronic hepatitis C without significant fibrosis.

## Introduction

Hepatitis C virus (HCV) infection is a major public health issue worldwide. The World Health Organization (WHO) has reported that HCV accounts for ~400,000 annual deaths globally, mostly due to end-stage complications of chronic liver disease, and vowed to eliminate HCV as a public health threat by the year 2030 [1]. This ambitious goal appears to be within reach thanks to the advent of potent and safe direct-acting antiviral-based regimens, resulting in a viral clearance in excess of 95% in most patients’ subgroups [2]. HCV clearance has been shown to be associated with the improvement of a wide array of clinical outcomes, such as hepatocarcinogenesis and liver-related mortality [3], but also with the restauration of innate immune responses [4] and an improved quality of life [5, 6].

An excess HCV-related morbidity and mortality has also been consistently reported to derive from several extrahepatic disorders, including insulin resistance (IR) and type 2 diabetes [7–10]. The causal relationship between HCV and glucose metabolic disturbances is supported by strong clinical and epidemiological evidence. Longitudinal studies have shown an excess of incident type 2 diabetes in patients with chronic hepatitis C, after adjustment for common risk factors, including elevated liver enzymes [11–13]. HCV clearance following antiviral therapy leads to reduced IR [14], reduced incidence of impaired glucose tolerance and type 2 diabetes, independently of other risk factors [15–17], reduced requirement of antidiabetic drugs in patients with diabetes [18, 19], reduced incidence of renal and cardiovascular complications [20–23] and of the associated mortality [24]. Chronic infection with hepatitis C genotype 3 is characterized by a distinct disease phenotype, including a moderate to severe liver steatosis, an accelerated liver fibrosis progression rate and an increased risk of hepatocellular carcinoma [25]. Despite the fact that the risk of diabetes in HCV infected patients is independent of the genotype [26], the clinical outcome in the genotype 3 is certainly worsen as compared to other genotypes. The pathogenesis of type 2 diabetes associated with HCV infection proceeds through IR. Patients with chronic hepatitis C have C-peptide and HOMA-IR levels significantly increased compared to individuals with chronic hepatitis B matched for age, sex and liver disease severity [27]. Since HCV infects primarily hepatocytes, it is intuitive to suggest a direct interaction between HCV proteins and the insulin signaling cascade inside hepatocytes. HCV-induced alterations of the insulin-mediated signal transduction have been reported in experimental models and human livers [28–30]. Nevertheless, recent works on HCV patients subjected to hyperinsulinemic-euglycemic clamp have suggested an indirect mechanism involving the presence of a significant extrahepatic component of IR, essentially located in skeletal muscles [26, 31, 32], implying undefined endocrine mediators secreted by infected hepatocytes. The missing piece of evidence should come from the reversal of these effects *via* a successful antiviral therapy. Treatment with IFNα-based regimens reduces the whole body IR [32, 33]. However, IFNα affects the insulin signaling transduction pathway *via* tyrosine phosphorylation of the insulin receptor substrate-1 [34], and may confound the interpretation of data. The recent approval of IFNα-free regimens [2] allowed us addressing this issue. Given the high prevalence of HCV infection worldwide, understanding the molecular mechanisms leading to the development of IR is of major interest and may provide working hypotheses to unravel the pathogenesis of type 2 diabetes.

In a single-arm exploratory trial, we evaluated the impact of an IFNα-free therapy on the level of hepatic *vs*. extrahepatic insulin sensitivity. The primary outcome of the study was to determine glucose consumption variation (as measured by euglycemic hyperinsulinemic clamp) in non-diabetic, lean chronic hepatitis C patients lacking significant fibrosis before and after complete suppression of viral replication.

## Materials and methods

### Trial design

The TREND check list as well as the protocol of the trial are available as supplementary information (S1 Checklist, S1 Protocol). The study was approved by the ethics committee of the Canton of Geneva and registered with ClinicalTrials.gov under the accession number NCT02760355. The selection of patients followed at the Gastroenterology and Hepatology Division of the Geneva University Hospitals started in 2016 and was performed during a routine consultation. Participants fulfilling all the following inclusion criteria were eligible for the study: adult Caucasian patient males or non-pregnant or non-lactating females chronically infected with HCV genotype 3 and 18–65 years at the time of the screening; lack of contraindications to the class of drugs under study, e.g. known hypersensitivity or allergy to class of drugs or the investigational products; lack of significant fibrosis or any feature of metabolic syndrome, two conditions which may impact glucose metabolism. Patients with excessive alcohol consumption (>30 g/day in males and >20 g/day in females), coinfection with human immunodeficiency virus or hepatitis B virus, concomitant medications interacting with the study drugs, or any significant medical condition potentially interfering with the adherence to the study procedure were excluded. Written informed consent was obtained from all subjects before entering the study.

Sample size calculation was based on the primary outcome i.e. an estimated difference in peripheral insulin sensitivity between two interventions of 10%, with a standard deviation of 6%, and it was determined a sample size of nine volunteers would be sufficient.

Patients fulfilling the inclusion criteria received a fixed-dose combination tablet containing 400 mg of sofosbuvir and 90 mg of ledipasvir once daily plus body weight-based ribavirin (i.e. 1,000 mg if <75 Kg or 1,200 mg if >75 Kg, in two daily doses), delivered either by FN or GG at the the Clinical Research Unit of Geneva University Hospitals. Although this regimen was not included in international treatment guidelines for HCV genotype 3, due to limitations that became evident at later stages, at the time of the trial set-up it seemed the most potent IFNα-free regimen to treat this particularly resistant viral genotype [35]. Serum HCV RNA levels were measured using the COBAS Ampliprep/COBAS TaqMan 2.0 assay (Roche Molecular Systems, Pleasanton, CA) with a lower limit of detection <15 IU/mL. Complete on-treatment virological response was defined as undetectable HCV RNA in serum 6 weeks from treatment start.

From March 2016 to May 2018, patients were submitted to two investigation visits (see below for detailed metabolic protocol) before and after six weeks of treatment. After sustained viral response (SVR), three out of the twelve patients participated to an additional clamp.

The day of each investigation, all data were collected and stored in an individual folder per patient. In each folder, the following data were collected in a paper Case Report Form: demographic data, visit dates, concomitant medication, biochemical and viral data (extracted from the electronic patient record of the Geneva University Hospitals), drug information provided by the pharmacy, results of the metabolic analyses, and adverse events. The essential documents according to the ICH GCP guidelines E6(R2) including the Informed consent forms were stored in the Trial Master File.

**Fig 1:**
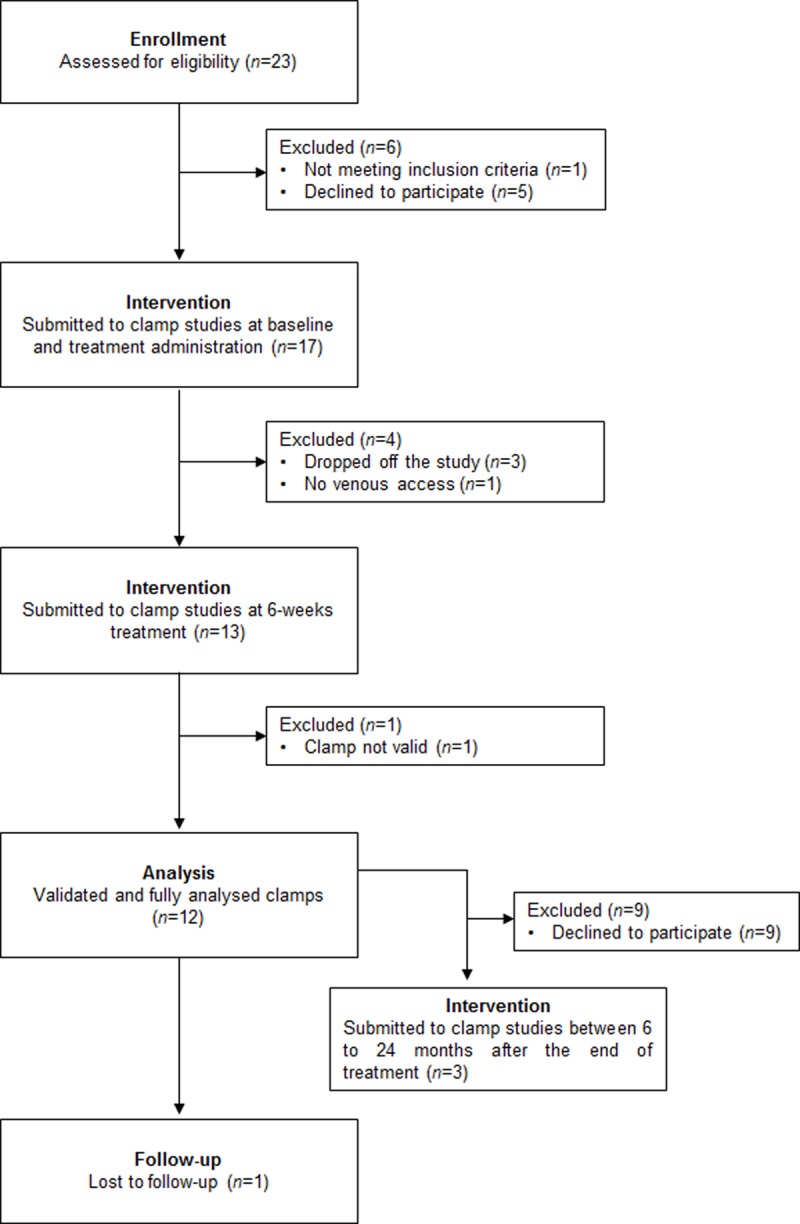
Flowchart of the study design.

Flowchart summarizing the study is depicted in Fig 1.

### Metabolic protocol

To assess the relative contribution of hepatic *vs*. extrahepatic tissues to the whole body insulin sensitivity, patients were asked to undergo a thorough investigation visit before and after six weeks of treatment, conducted by GG at the Clinical Research Unit of Geneva University Hospitals. For both visits, patients were admitted to the hospital in the morning, after an overnight fast (>10h). Body weight, height, waist and hip circumference, blood pressure were measured. An adipose tissue biopsy was obtained [36].

Insulin sensitivity was assessed by performing a two-step hyperinsulinemic euglycemic clamp (0.3 mU · kg−1 · min−1 and 1 mU · kg−1 · min−1 for 90 min each) with non-radioactive tracers ([6,6]-^2^H_2_-glucose and ^2^H_5_-glycerol, Cambridge Isotope Laboratories, Innerberg, Switzerland) [37] (S1 Fig). Blood samples were collected at 30 min intervals for the analysis of tracers, non-esterified fatty acids (NEFA), glucose and insulin and every 5 min during the clamp to control plasma glucose concentration, which was maintained constant at ∼5 mmol/L by infusing a 20% (w/v) glucose solution. Total glucose rate of appearance, endogenous glucose production rate (EGP) and glycerol rate of appearance (glycerol Ra) were calculated [38]. Respiratory gas exchanges were monitored for the last 30 min of the different steps of the clamp by open-circuit indirect calorimetry [39]. The instrument was calibrated according to standard procedures.

Regional soft tissue composition were assessed at the femur (thigh), D12 and L5 level using low dose unenhanced computed tomography (CT). Edge-detection and threshold techniques were used to separate tissues (i.e. adipose, muscle and bone) based on attenuation characteristics, which are directly related to tissue composition and density [40, 41]. To measure subcutaneous and visceral fat, single (1 cm) CT scan slices were obtained at the level of umbilicus and at level of the thigh 15 cm from the greater trochanter. CT scans were analyzed using density contour software (Osirix MD, v.9.0.2, Pixmeo, Switzerland). Adipose tissue was classified according to a density of -250 to -50 Hounsfield units. Visceral fat area (within the borders of the *fascia transversalis*), total thigh area and thigh subcutaneous fat area of the two legs were measured and the thigh muscle area was calculated as the difference between total thigh area of both legs and thigh subcutaneous fat area. Change in area was calculated as the difference between the values obtained before and after 6 weeks of treatment [42].

### Laboratory assays

Plasma glucose and NEFA concentrations were determined by a colorimetric method (RX Monza, Randox Laboratories Ltd, United Kingdom). Plasma insulin was measured by RIA (EMD Millipore, St. Louis, Missouri, USA). Plasma [6,6-2H2]glucose and ^2^H_5_-glycerol concentration were measured by gas chromatography-mass spectrometry [43].

### Quantification of plasma cytokines

Levels of 27 cytokines and other proteins involved in metabolism or inflammation were measured in plasma before and after 6 weeks of antiviral treatment, using either a customized bead-based multiplex (Human magnetic luminex assay, R&D systems) or by commercial ELISA (S1 Table). Samples and standards were run in duplicate.

### RNA extraction and real-time RT-PCR

Total RNA was extracted using the NucleoSpin RNA set for NucleoZOL kit (Macherey-Nagel, Düren, Germany). cDNA was synthesized from 500 ng total RNA with Transcriptor Universal cDNA master (Roche Diagnosis, IN). For real-time PCR, the following primers were used: hormone-sensitive lipase (LIPE, forward ACGGTGGCCGATGCCATGTT and reverse AGCTGCGTGGGGCTGAGTTT) and adipose triglyceride lipase (ATGL, forward GTGTCAGACGGCGAGAATG and reverse TGGAGGGAGGGAGGGATG). Relative quantification was performed by real-time PCR as described [44].

### Histology and morphometry

Adipose tissue biopsies were formalin-fixed, paraffin-embedded and processed for H&E staining. Lipid droplet area was calculated using Definiens software (Definiens AG, Munich, Germany).

### Statistical analysis

Statistical analysis was performed using Graphpad Prism 7 software. All results were expressed as means ±SD and analysed by paired t-test. Correlation analyses were done by using the Pearson’s correlation coefficient test A value of *p*<0.05 was considered significant. To account for multiple testing, we corrected the p-value by calculating the false discovery rate (FDR) (Benjamini-Hochberg method) and using a cut-off value of FDR less than 0.1 to be significant.

## Results

### Characteristics of the study population

Seventeen non-diabetic, lean chronic hepatitis C patients infected with HCV genotype 3 and without significant fibrosis, as determined by the Metavir score at liver biopsy or by transient elastography (Fibroscan^TM^) were included in the study (Table 1).

**Table 1:** Characteristics of the study population (n = 12).

Patient no.	Sex (M/F)	Age (y)	Opiate-substitution therapy (yes/no)	Liver fibrosis	ALAT /ASAT (U/L)	Fasting glucose (mM)	Fasting insulin (μU/Ml)	HOMA-IR	HbA1c % (mmol/mol)
				(stage F by Metavir or kPa by Fibroscan^TM^)					
1	F	37	no	3.5±0.8	33/25	5.2	11.2	2.6	5.1 (32)
2	F	44	no	4.4±1.2	28/24	5.4	10.3	2.5	4.8 (29)
3	M	55	no	F0	44/30	5.5	4.9	1.2	5.2 (33)
4	M	33	no	F1	82/37	4.4	9	1.8	4.9 (30)
5	F	56	no	3.8±2.9	202/135	6	25.8	6.9	5.4 (36)
6	M	24	yes	F1	45/32	5	7.6	1.7	5.0 (31)
7	M	55	no	5.3	97/49	6.2	14.6	4	5.4 (36)
8	M	55	no	5.8	78/56	5.3	12.7	3	5.4 (36)
9	F	59	no	F0	32/31	5.1	11	2.5	5.1 (32)
10	F	42	no	F0	137/107	5.2	11.6	2.7	4.8 (29)
11	M	56	no	F0	206/94	4.5	16.6	3.3	5.1 (32)
12	M	38	no	F1	66/39	5.3	8.5	2	5.4 (36)

Abbreviations: HOMA-IR, homeostatic model assessment of insulin resistance; HbA1c, glycated hemoglobin.

Out of the 17 patients enrolled, 13 (8 males and 5 females) completed the study and underwent both hyperinsulinemic euglycemic clamps (Fig 1). During the visit at baseline, one patient was not properly clamped with insulin and thus excluded from the analysis. Subjects had a median age of 49.5 years [24–59] (Table 1).

All the 12 patients adhered to the treatment and reached complete virological response at 6 weeks (Table 2). No important adverse effects were reported.

**Table 2:** Subjects characteristics at baseline after 6 weeks of antiviral treatment (n = 12).

Variable (*n* = 12)	Baseline	6-week treatment	*p*
BMI (kg/m^2^)	23.0 ± 2.9	23.0 ± 2.9	0.999
Body weight (Kg)	67 ± 12.9	67.07 ± 13	0.815
Waist circumference (cm)	83.9 ± 13.3	78.2 ± 16.1	0.105
Systolic BP (mmHg)	121.4 ± 15.5	120.8 ± 13.9	0.881
Diastolic BP (mmHg)	76.6 ± 13.3	75.7 ± 11.5	0.775
**Glucose metabolism**			
Fasting glucose (mM)	5.3 ± 0.5	5.3 ± 0.5	0.685
Fasting insulin (μU/mL)	12.0 ± 5.3	12.0 ± 4.0	0.977
**Liver enzymes**			
ASAT (U/L)	54.9 ± 36.7	23.1 ± 2.4	**0.013**
ALAT (U/L)	87.5 ± 62.9	21.9 ± 4.6	**0.003**
Alkaline phosphatase (U/L)	59.9 ± 23.0	59.5 ± 20.0	0.879
GGT (U/L)	37.0 ± 21.6	17.6 ± 8.2	**0.001**
**Lipids**			
Cholesterol (mmol/L)	4.2 ± 0.6	5.0 ± 1.1	**0.001**
TG (mmol/L)	1.4 ± 0.6	1.3 ± 0.7	0.776
LDL (mmol/L)	2.1 ± 0.6	2.8 ± 0.9	**0.0006**
HDL (mmol/L)	1.5 ± 0.37	1.6 ± 0.6	0.293
Subcutaneos fat volume	232.6 ± 118.5	228.7 ± 114.3	0.385
Visceral fat volume	84.3 ± 67.3	85.83 ± 70.4	0.447
**HCV RNA Log10 (IU/mL)**	6.4 ± 6.6	<1.2	-

Abbreviations: BMI, body mass index; ASAT, aspartate aminotransferase; ALAT, alanine aminotransferase; GGT, gamma glutamyl transpeptidase; TG, triglycerides; LDL, low-density cholesterol; HDL, high density cholesterol. Data are means ± SD.

The complete suppression of viral replication was accompanied by a significant decrease of serum ASAT, ALAT and GGT, but also by an expected, significant increase of total and LDL cholesterol [45] (Table 2). No changes were observed in body weight, body mass index, blood pressure, or visceral/subcutaneous fat volume (Table 2 and S2 Fig).

### Glucose metabolism

**Fig 2:**
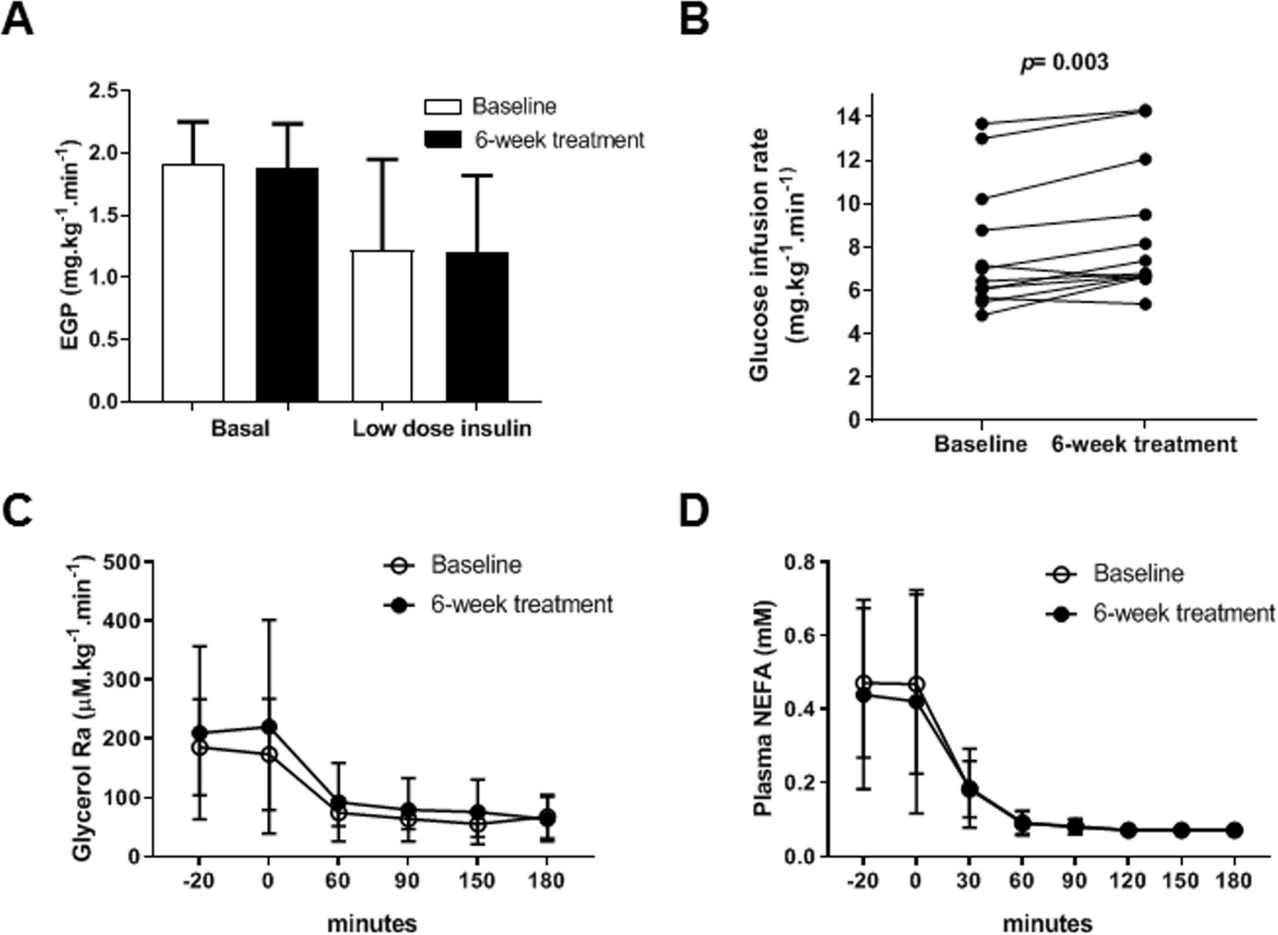
Euglycemic hyperinsulinemic clamp. (A-B) Glucose metabolism. (C-D) Lipid metabolism. Endogenous glucose production (EGP) at baseline (open bars) and after 6 weeks of antiviral treatment (black bars) in the basal state and during low-dose insulin infusion rate (0.3 mU.kg^-1^.min^-1^). Data are means ± SD. **(B)** Glucose infusion rate during high-dose insulin infusion rate (1 mU.kg^-1^.min^-1^) at baseline and after 6 weeks of antiviral treatment (**p = 0.003). Insulin-mediated lipolysis suppression at baseline and 6-week treatment measured by **(C)** glycerol tracer (*n* = 8) and by **(D)** non-esterified fatty acid (NEFA) levels in plasma (*n* = 12), in basal state and clamp conditions. Data are means ± SD.

Basal levels of plasma glucose and insulin were similar as measured before and after 6 weeks of treatment (Table 2). Under clamp conditions, insulin levels increased to similar steady-state levels in both clamp experiments, while normoglycemia (plasma glucose 5.0 mmol/L) was maintained (S3 Fig). Hepatic insulin sensitivity was estimated by calculating endogenous glucose production (EGP), which corresponds to the difference between glucose rate of appearance and exogenous glucose infusion rate. Low-dose insulin infusion rate induced EGP suppression as compared to basal state, however no difference was observed in EGP between before and after 6 weeks of treatment, under both basal and clamp conditions (p = 0.70 and 0.90, respectively) (Fig 2A). At high-dose insulin infusion rate, EGP was completely suppressed. Peripheral insulin sensitivity was assessed by measuring glucose infusion rate during high-dose insulin. Ten out of 12 patients exhibited a significantly increased glucose infusion rate after 6 weeks of treatment compared to baseline (median [range]: 13.1% [4.6–36.7]) (p = 0.003) (Fig 2B), indicating that HCV suppression led to an improved peripheral insulin sensitivity. However, no significant alterations were found in oxidative and nonoxidative glucose, measured by indirect calorimetry between before and after 6 weeks of treatment (S2 Table).

To confirm the improved insulin sensitivity, we performed a third clamp in three patients who agreed to undergo the procedure, at least 6 months after the end of treatment. Two patients who permanently cleared HCV maintained an increased glucose infusion rate (+8% and +18%, respectively) with respect to pre-treatment levels. On the contrary, the peripheral insulin sensitivity of the third patient who experienced a relapse in HCV infection returned to baseline levels, consistent with the hypothesis that HCV directly induces this metabolic effect.

Transaminase levels are a proxy for liver inflammation. Thus, we assessed whether the decline of ALAT levels would be correlated with the improvement of glucose infusion rate, which provides a measure of peripheral insulin sensitivity. We found no correlation between glucose infusion rate changes and ALAT decrease (r = -0.177, p = 0.58), suggesting that the pathogenesis of peripheral IR induced by HCV in patients with mild liver damage may proceed independently of liver inflammation.

### Lipid metabolism

Lipid metabolism was studied by determining insulin-mediated lipolysis suppression. In adipose tissue, insulin suppresses triglyceride hydrolysis into glycerol and NEFA, and induces lipogenesis leading to an increased energy storage. As expected, low-dose insulin infusion induced a decrease of glycerol Ra and plasma levels of NEFA (Fig 2C and 2D, respectively). At high-dose insulin infusion rate, lipolysis was completely suppressed. No significant variation was observed between baseline and after 6 weeks of treatment in insulin-mediated lipolysis suppression, both under basal and clamp conditions (Fig 2C and 2D). In accordance, the expression of genes in adipose tissue involved in lipolysis (*LIPE* and *ATGL*) was unchanged as compared to pre-treatment (S4A Fig). No effect of viral suppression on lipid oxidation was observed (S2 Table). Adipose tissue histology and lipid droplet morphology and size were not modified upon treatment (S4B Fig). These data suggest that HCV does not affect lipid homeostasis in adipose tissue, contrary to what was observed in the liver [46, 47].

### Antiviral treatment affects cytokine profile

**Fig 3:**
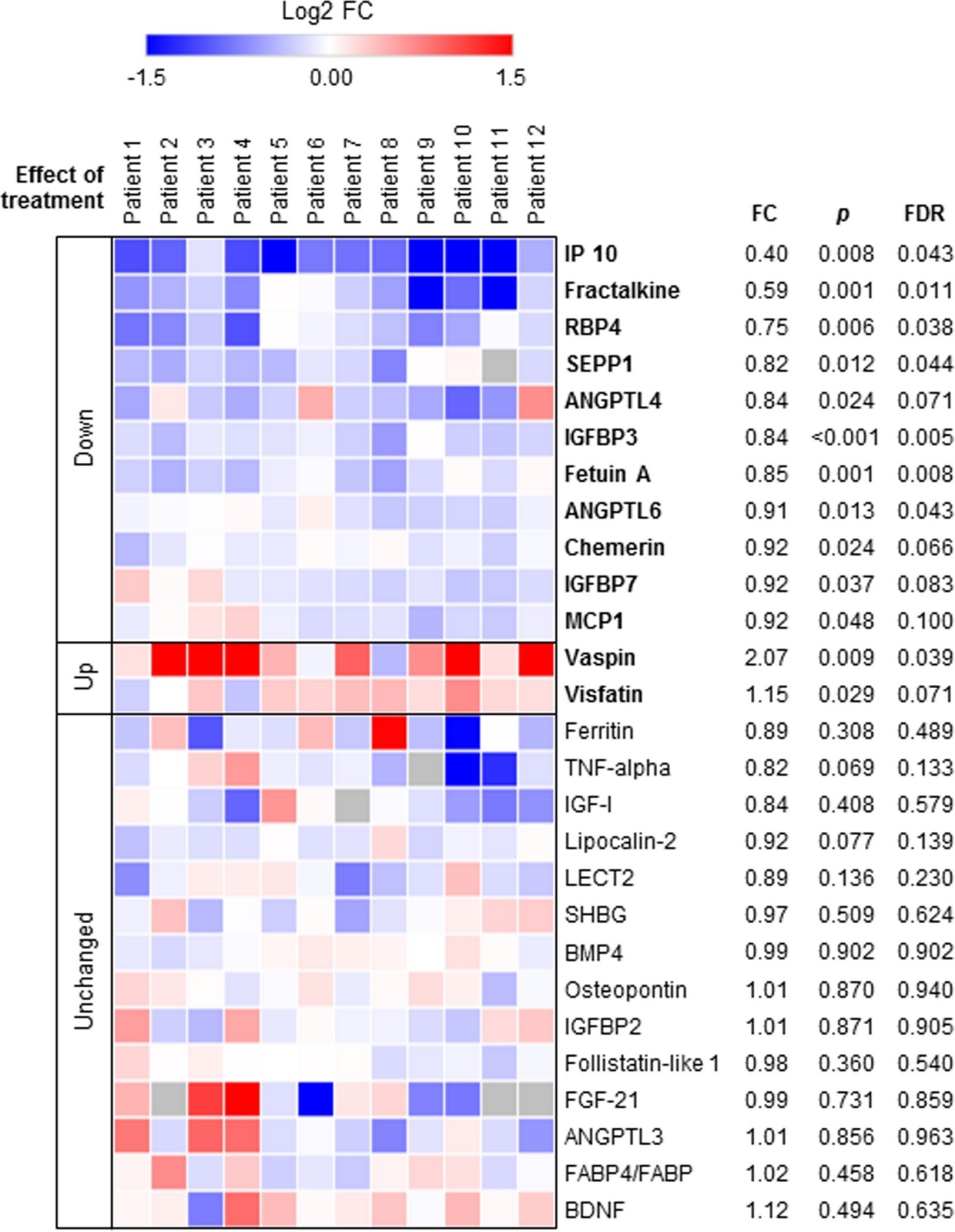
Heat map of 27 cytokines and other metabolically relevant proteins determined in plasma of 12 chronic hepatitis C patients before and after 6 weeks of antiviral treatment. Data are shown as Log-2 fold change to baseline levels. Each row and column represents a specific cytokine and patient, respectively. Blue and red colors indicate cytokines found to be down-regulated and up-regulated, respectively, after treatment. FC, fold change after 6 weeks of treatment compared to pretreatment values. P-value were corrected for multiple testing by calculating the false discovery rate (FDR). A cut-off value of FDR less than 0.1 was considered to be significant.

The plasma levels of 27 cytokines and other factors were measured at baseline and after 6 weeks of antiviral treatment in all patients. The levels of some of them were significantly modified upon viral suppression: compared to baseline, the levels of IP10, fractalkine, retinol binding protein 4 (RBP4), selenoprotein P (SEPP1), angiopoietin-like-4 (ANGPTL-4) and -6 (ANGPTL-6), insulin-like growth factor-binding protein 3 (IGFBP-3) and -7 (IGFBP-7), fetuin-A, chemerin, and MCP-1 were decreased, while those of visfatin and vaspin were increased after 6 weeks of treatment (Fig 3). Of note, we did not detect significant changes in the circulating levels of TNFα (Fig 3).

## Discussion

In this study, we show that (i) the complete suppression of HCV replication induced by an IFNα-free regimen in lean chronic hepatitis C patients without significant fibrosis significantly improves the extrahepatic (but not the hepatic) insulin sensitivity, (ii) the improved glucose homeostasis bears no correlation with the transaminase decline, suggesting the HCV-induced extrahepatic IR and liver inflammation are probably disconnected from each other, (iii) HCV does not seem to interfere with the insulin effects on lipid metabolism in adipose tissue, and (iv) HCV modifies the circulating levels of factors likely involved in the pathogenesis of the decreased peripheral insulin sensitivity.

The improvement in HCV-induced glucose metabolic alterations following antiviral therapy has been initially reported using IFNα-based regimens [30]. These data should be taken with caution, due to the known effects of IFNα on the insulin signaling pathway [25] and on body weight. Recently, improved glucose homeostasis has been reported also in patients treated with IFNα-free regimens, and patients with diabetes may even require reduced amounts of medicines to maintain glycemic control [18, 19, 48, 49]. Such studies have measured the improvement of routine parameters of glucose metabolism, such as glycated hemoglobin or HOMA-IR scores, thus falling short of analyzing in detail the relative contribution of the liver *vs*. extrahepatic organs to the whole insulin sensitivity. A single study, using a clamp assessment, assessed patients treated with an IFNα-based regimen [32]. Here, for the first time we measured the hepatic and peripheral insulin sensitivity in patients treated with an IFNα-free regimen. In addition, we also chose to assess the insulin sensitivity at the time of complete suppression of viral replication rather than upon consolidation of SVR, because the latter may be associated with unpredictable changes in body weight [50].

Our data are in agreement with two previous independent studies in chronic hepatitis C patients without stigmata of the metabolic syndrome [26, 31]. These were the first to report a significant extrahepatic component of viral IR, based on euglycemic hyperinsulinemic clamp measurements. However, only one of those studies [26] reported a hepatic component of the whole body IR, while the other one [31] merely suggested a possible, small hepatic contribution. In a follow-up study [32], clamps performed before and after IFNα-induced viral clearance could not identify with certitude a hepatic IR. It is possible that the hepatic IR shown in the initial work by Vanni *et al*. [26] may be due to a higher degree of hepatic inflammation and fibrosis, while all our patients lacked significant fibrosis. In our study, the reduction in the glucose infusion rate during clamp at the time of complete viral suppression failed to correlate with the improvement of ALAT levels, a proxy for liver inflammation. In addition, we failed to detect significant changes in the circulating levels of TNFα, an inflammatory cytokine increased in chronic hepatitis C [26, 31, 51], especially with type 2 diabetes [52]. Taken together, these data suggest that the pathogenesis of peripheral IR induced by HCV in patients with mild liver damage may proceed independently of liver inflammation and fibrosis. A significant inflammation may however accelerate this process, as suggested by experimental [53] and clinical data [13].

We also measured the circulating levels of several circulating factors which may influence metabolic processes. For many of them, levels were unchanged upon treatment (Fig 3), suggesting that they may not have any significant impact on HCV-induced IR. For others, changes induced by antivirals deserve a comment, as they may be directly or indirectly involved in the pathogenesis of HCV-related IR.

Nine liver-secreted factors were dysregulated in viremic patients: fractalkine, RBP4, SEPP1, fetuin-A, IGFBP-3, IGFBP-7, chemerin and ANGPTL-4 and -ANGPTL-6. The plasma levels of all these factors were significantly decreased upon suppression of the viral replication, suggesting that HCV infection leads to their upregulation.

Fractalkine is a chemoattractant to T cells and monocytes, and may be a promoter of systemic inflammation, although its role in diabetes is controversial [54]. In hepatitis C, it is associated with liver disease severity and fibrosis progression [55, 56].

RBP4, a retinol transporting protein from liver to peripheral tissues, is upregulated in HCV-infected persons *vs*. healthy controls [57]. *In vitro*, HCV stimulates RBP4 expression, while RBP4 knockdown increases HCV replication, suggesting that RBP4 upregulation may be a mechanism of viral attenuation or an adaptive host response to infection. RBP4 increases gluconeogenesis by stimulating phosphoenolpyruvate kinase and impairs insulin signaling in the muscle, leading to IR [58]. Indeed, RBP4 is an important biomarker for several metabolic disorders [59].

SEPP1, a selenium transporter, impairs insulin signaling by inhibiting insulin-stimulated glucose uptake in myotubes [60]. SEPP1 is increased in chronic hepatitis C patients with diabetes *vs*. non-diabetic infected controls [61].

Fetuin-A is an important independent risk factor for the development of type 2 diabetes [62], to the point that it has been suggested as a therapeutic target [59]. Its levels are increased in patients with IR [63], including in chronic hepatitis C [61].

IGFBP-3 must be assessed as ratio IGF-1/IGFBP-3, reported for being inversely correlated with IR [64]. In our work, IGF-I levels were unchanged, translating into an increased IGF-I/IGFBP-3 *ratio*, consistent with the improved insulin sensitivity. Similarly, IGFBP-7 was reported to be highly expressed in patients with IR [65].

Finally, chemerin, expressed in the adipose tissue but also in the liver, makes skeletal muscle cells insulin resistant, inducing a markedly decrease glucose uptake [66]. Systemic levels are elevated in chronic hepatitis C [67].

As to ANGPTL-4 and ANGPTL-6, their role in glucose homeostasis is less clear. In humans, ANGPTL4 levels are inversely correlated with glycemia and HOMA-IR, while in patients with type 2 diabetes, plasma levels of ANGPTL4 are significantly lower than in healthy subjects [68]. Also ANGPTL-6 is playing a protective effect by antagonizing obesity and IR. In humans, plasma concentrations were increased in diabetic *vs*. non-diabetic subjects [69]. The changes observed in our work suggest that may be part of a host adaptive response to the glucose metabolic alterations induced by HCV.

Vaspin and visfatin levels increased significantly upon viral suppression. Vaspin is an adipokine elevated in obesity and type 2 diabetes [70]. Administration of vaspin improves glucose tolerance in obese mice [71]. In humans, it increases after physical exercise [72], while *in vitro* it has insulin-sensitizing effects [73]. Experimental data indicate that vaspin may be a host compensatory response to decreased insulin sensitivity. The fact that HCV suppression leads to increased vaspin levels suggests that the viral IR may be also mediated by blockade of host adaptive responses. Since vaspin is secreted by adipocytes, this blockade seems indirect, again suggesting a cross-talk between infected and uninfected tissues. Visfatin is another adipokine that stimulates insulin signaling [74], and its increase upon viral suppression is consistent with an increased insulin sensitivity.

Thus, we identified a profile of cytokines likely involved in HCV-induced IR. Treatment-induced viral suppression led to a decrease of circulating levels of cytokines promoting IR (fractalkine, RBP4, SEPP1, fetuin-A, IGFBP-3, IGFBP-7 and chemerin) and to an increase of two adipokines involved in protection from IR (vaspin and visfatin). The molecular mechanisms leading to this altered profile in viremic patients may be direct (for hepatokines) or indirect (for factors not expressed by hepatocytes). These results provide a rationale for studying the details of the liver-to-periphery cross-talk leading to HCV-induced IR, as shown by our clamp data after treatment-induced viral suppression.

Although the burden of hepatitis C worldwide is dwindling due to the advent of potent antivirals and the implementation of national strategies for viral elimination, our findings are still relevant and worth being studied in detail to provide working hypotheses to analyze the physiopathology of glucose homeostasis in other settings, such as non-alcoholic fatty liver disease, type 2 diabetes, and energy homeostasis.

## Supporting information

S1 Checklist (PDF)

S1 Table Bead-based multiplex and commercial ELISA kits. (DOCX)

S2 Table Indirect calorimentry data of 12 chronic hepatitis C patients before and after 6 weeks of treatment. (DOCX)

S1 Fig Scheme of the euglycemic hyperinsulinemic clamp design. (DOCX)

S2 Fig Computed tomography (CT)-scan representative images **(A)**, and fat and muscle volume quantification at baseline and after 6 weeks of treatment **(B)**.
(DOCX)

S3 Fig Plasma concentrations of glucose **(A)** and insulin **(B)** during euglycemic hyperinsulinemic clamp, at baseline and 6-week treatment.
(DOCX)

S4 Fig (A) mRNA levels of genes involved in lipolysis. **(**B) H&E-stained adipose tissue biopsies at baseline (a) and 6-week treatment (b) and quantification of the mean area of lipid droplets.
(DOCX)

S1 Protocol (PDF)
