# Supplementary material for: Treatment with direct-acting antivirals improves peripheral insulin sensitivity in non-diabetic, lean chronic hepatitis C patients
Source: PLoS One. 2019 Jun 6;14(6):e0217751. doi: 10.1371/journal.pone.0217751 (PMC6553748; doi:10.1371/journal.pone.0217751)
Supplement: S1 Table — (DOCX) [file pone.0217751.s002.docx]

**S1 table.** Bead-based multiplex and commercial ELISA kits

| **Cytokine** | **Kit** | **provider** | **ref** |
| --- | --- | --- | --- |
| Custom Multiplex | Human Magnetic Luminex Assay  *ANGPTL3, ANGPTL4, ANGPTL6, BDNF, BMP-4, CCL2/MCP-1, Chemerin, CX3CL1/Fractalkine, CXCL10/IP-10, FABP4/A-FABP, Fetuin A/AHSG, Follistatin-like 1, Ferritin, IGFBP-2, IGFBP-3, IGFBP-rp1/IGFBP-7, Lipocalin-2/NGAL, Osteopontin, Serpin A12/Vaspin, SHBG, RBP4, TNF-alpha* | R&D | - |
| Fibroblast growth factor-21 (FGF-21) | Human FGF-21 ELISA | BioVendor | RD191108200R |
| Leukocyte cell–derived chemotaxin 2 (LECT2) | Human LECT-2 ELISA | BioVendor | RD191370200R |
| Insulin like growth factor-1 (IGF-I) | Human IGF-1 ELISA | BioVendor | RMEE20 |
| selenoprotein P (SEPP1) | Human SEPP1 ELISA | Cusabio technology | CSBCSB-EL021018HU |
| Visfatin | Human visfatin ELISA | Phoenix Pharmaceuticals Inc. | EK-003-80 |
