# Supplementary material for: Treatment with direct-acting antivirals improves peripheral insulin sensitivity in non-diabetic, lean chronic hepatitis C patients
Source: PLoS One. 2019 Jun 6;14(6):e0217751. doi: 10.1371/journal.pone.0217751 (PMC6553748; doi:10.1371/journal.pone.0217751)
Supplement: S2 Table — (DOCX) [file pone.0217751.s003.docx]

**S2 table.** Indirect calorimentry data of 12 chronic hepatitis C patients before and after 6 weeks of treatment

| **Indirect calorimetry (mg^-1^.kg^-1^.min^-1^)** | **Baseline** | **After 6 weeks of treatment** | ***p*** |
| --- | --- | --- | --- |
|  |  |  |  |
| **Basal** | | | |
| Lipid oxidation | 1.17 ± 0.3 | 1.13 ± 0.4 | 0.802 |
| **Low-dose insulin infusion rate** | | | |
| Lipid oxidation | 0.87 ± 0.2 | 0.88 ± 0.3 | 0.866 |
| Glucose oxidation | 1.37 ± 0.8 | 1.51 ± 1.0 | 0.542 |
| **High-dose insulin infusion rate** | | | |
| Lipid oxidation | 0.71 ± 0.2 | 0.74 ± 0.3 | 0.703 |
| Glucose oxidation | 1.99 ± 0.7 | 2.22 ± 0.9 | 0.135 |
| Nonoxidative glucose | 5.87 ± 2.5 | 6.47 ± 2.5 | 0.053 |

Data are expressed as means ± SD.
