# Supplementary figures and images for: Treatment with direct-acting antivirals improves peripheral insulin sensitivity in non-diabetic, lean chronic hepatitis C patients
Source: PLoS One. 2019 Jun 6;14(6):e0217751. doi: 10.1371/journal.pone.0217751 (PMC6553748; doi:10.1371/journal.pone.0217751)

**
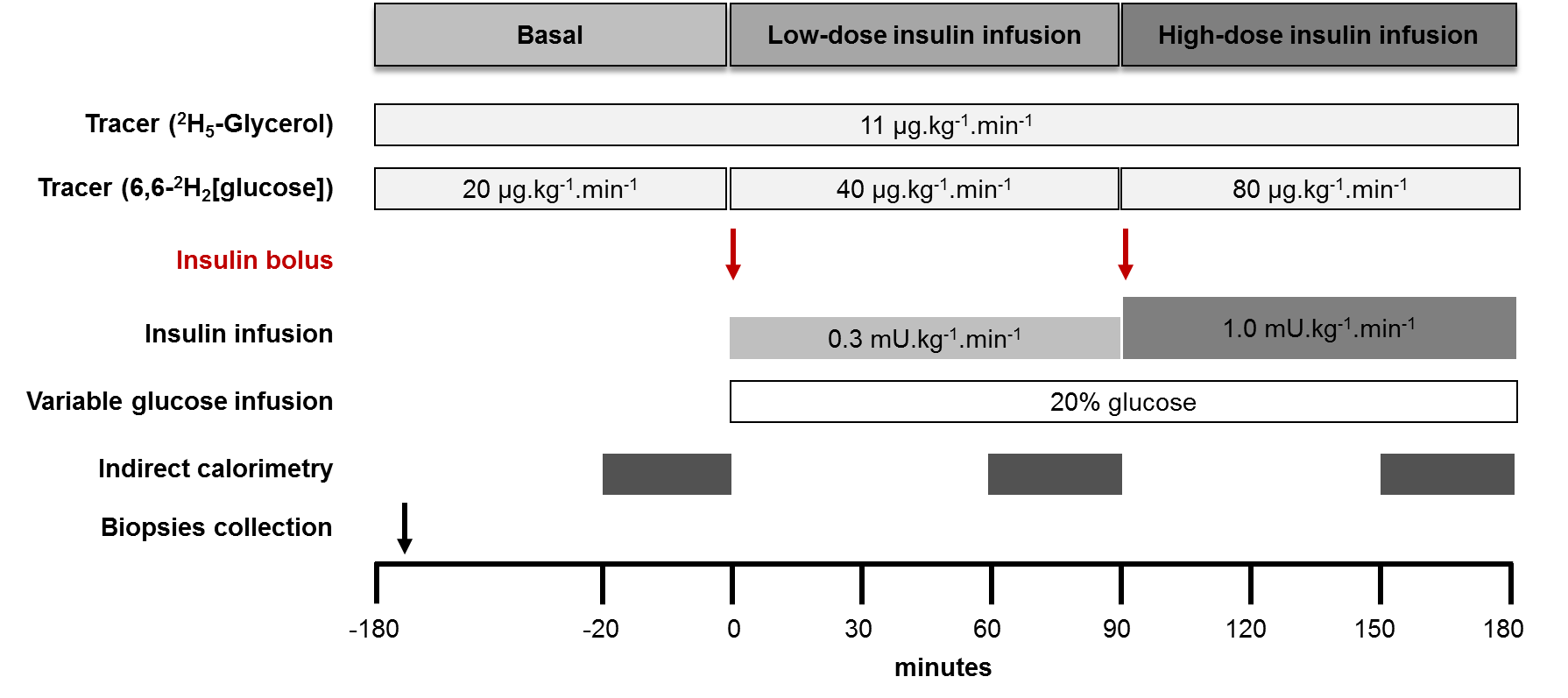
**

**S1 Fig.** Scheme of the euglycemic hyperinsulinemic clamp design

Supplement: S1 Fig — (DOCX) [file pone.0217751.s004.docx]
