# Supplementary material for: Treatment with direct-acting antivirals improves peripheral insulin sensitivity in non-diabetic, lean chronic hepatitis C patients
Source: PLoS One. 2019 Jun 6;14(6):e0217751. doi: 10.1371/journal.pone.0217751 (PMC6553748; doi:10.1371/journal.pone.0217751)
Supplement: S2 Fig — Computed tomography (CT)-scan representative images (A), and fat and muscle volume quantification at baseline and after 6 weeks of treatment (B). (DOCX) [file pone.0217751.s005.docx]

**
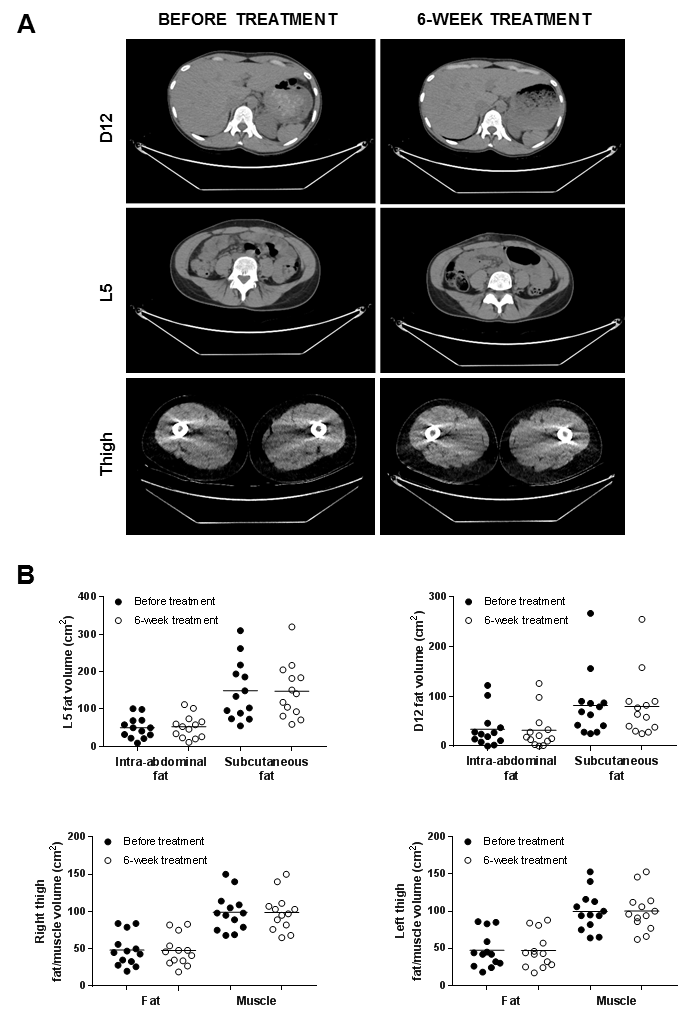
**

**S2 Fig.** Computed tomography (CT)-scan representative images **(A)**, and fat and muscle volume quantification at baseline and after 6 weeks of treatment **(B)**.
