# Supplementary material for: Treatment with direct-acting antivirals improves peripheral insulin sensitivity in non-diabetic, lean chronic hepatitis C patients
Source: PLoS One. 2019 Jun 6;14(6):e0217751. doi: 10.1371/journal.pone.0217751 (PMC6553748; doi:10.1371/journal.pone.0217751)
Supplement: S3 Fig — Plasma concentrations of glucose (A) and insulin (B) during euglycemic hyperinsulinemic clamp, at baseline and 6-week treatment. (DOCX) [file pone.0217751.s006.docx]

**
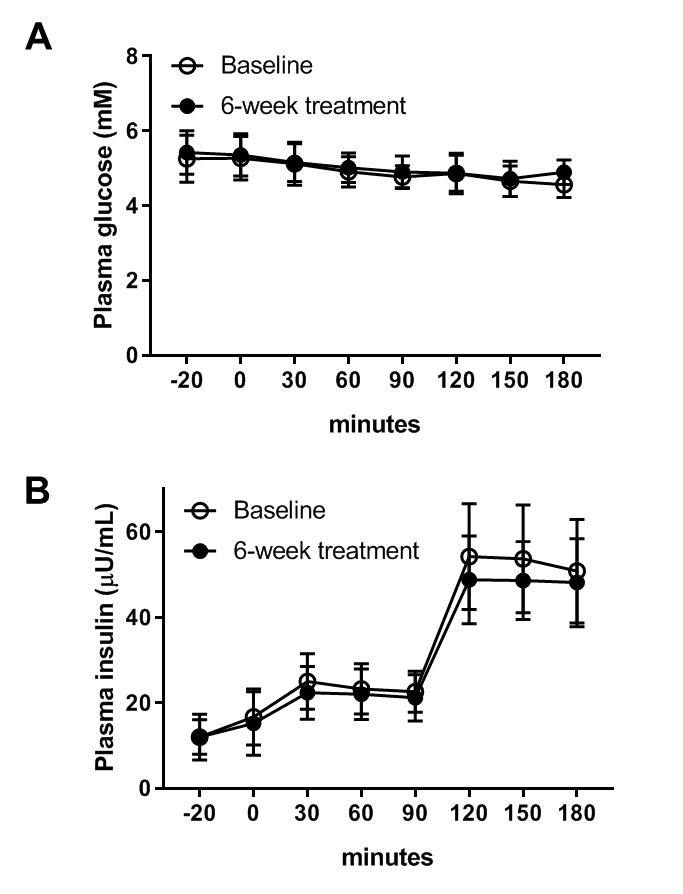
**

**S3 Fig.** Plasma concentrations of glucose **(A)** and insulin **(B)** during euglycemic hyperinsulinemic clamp, at baseline and 6-week treatment
