# Supplementary material for: Treatment with direct-acting antivirals improves peripheral insulin sensitivity in non-diabetic, lean chronic hepatitis C patients
Source: PLoS One. 2019 Jun 6;14(6):e0217751. doi: 10.1371/journal.pone.0217751 (PMC6553748; doi:10.1371/journal.pone.0217751)
Supplement: S4 Fig — (A) mRNA levels of genes involved in lipolysis. (B) H&E-stained adipose tissue biopsies at baseline (a) and 6-week treatment (b) and quantification of the mean area of lipid droplets. (DOCX) [file pone.0217751.s007.docx]

**
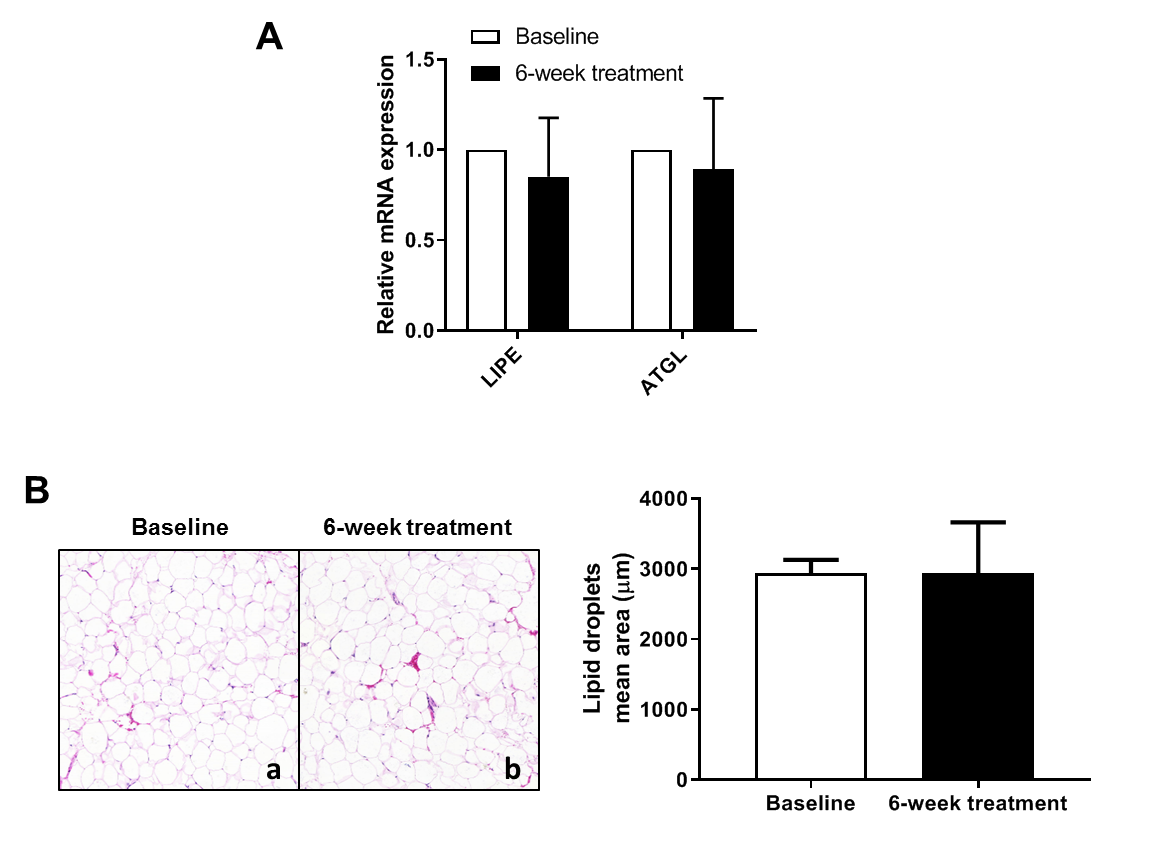
**

**S4 Fig. (A)** mRNA levels of genes involved in lipolysis. **(B)** H&E-stained adipose tissue biopsies at baseline (a) and 6-week treatment (b) and quantification
