# Supplementary material for: Treatment with direct-acting antivirals improves peripheral insulin sensitivity in non-diabetic, lean chronic hepatitis C patients
Source: PLoS One. 2019 Jun 6;14(6):e0217751. doi: 10.1371/journal.pone.0217751 (PMC6553748; doi:10.1371/journal.pone.0217751)
Supplement: S1 Protocol — (PDF) [file pone.0217751.s008.pdf]

Services de Gastroentérologie et
d'hépatologie et de Pathologie clinique

Hôpitaux Universitaires Genève

Rue Gabrielle-Perret-Gentil 4
CH – 1211 Genève 14

Hepatitis C virus infection induced insulin resistance: different contribution from liver and extrahepatic sites as inferred by treating chronic hepatitis C patients with an interferon-free antiviral combination

Clinical Study Protocol

Study Type:	Clinical trial with Medicinal Products (MP) authorised in Switzerland for genotype 1 infection but used in this clinical trial on genotype 3 infection (MP already approved in Europe for this application on genotype 3 infection)
Study Categorisation:	Risk category according to LHR : B
Study Registration:	Clinicaltrials.gov and FOPH portal
Study Identifier:	CCER 15-063
Principal Investigator &:<br>Sponsor Representative	Prof. Francesco Negro, Services de Gastroentérologie et d'hépatologie et de Pathologie clinique, Hôpitaux Universitaires de Genève, rue Gabrielle-Perret-Gentil 4, 1211 Genève 14.
Sponsor :	University Hospital Of Geneva (HUG)<br>rue Gabrielle-Perret-Gentil 4, 1211 Genève 14.
Investigational Product:	HARVONI® (Ledipasvir 90 mg/Sofosbuvir 400 mg) – Ribavirin
Protocol Version and Date:	V 2 of 27.07.2015

CONFIDENTIAL

The information contained in this document is confidential and the property of Prof. Francesco Negro-HUG (or "sponsor"). The information may not - in full or in part - be transmitted, reproduced, published, or disclosed to others than the applicable Competent Ethics Committee(s) and Regulatory Authority(ies) without prior written authorisation from the sponsor except to the extent necessary to obtain informed consent from those who will participate in the study.

Services de Gastroentérologie et
d'hépatologie et de Pathologie clinique

Hôpitaux Universitaires Genève

Rue Gabrielle-Perret-Gentil 4
CH – 1211 Genève 14

Signature Page(s)

Study number	CCER: 15-063
Study Title	Hepatitis C virus infection induced insulin resistance: different contribution from liver and extrahepatic sites as inferred by treating chronic hepatitis C patients with an interferon-free antiviral combination

The Sponsor-Investigator and trial statistician have approved the protocol **version 2 – 27 July 2015**, and confirm hereby to conduct the study according to the protocol, current version of the World Medical Association Declaration of Helsinki, ICH-GCP guidelines and the local legally applicable requirements.

Sponsor-Investigator:

Prof. Francesco Negro

Geneva, 27 juillet 2015

Place/Date Signature

Co-Investigator :

I have read and understood this trial protocol and agree to conduct the trial as set out in this study protocol, the current version of the World Medical Association Declaration of Helsinki, ICH-GCP guidelines and the local legally applicable requirements.

Co-Investigator:

Dr Giacomo Gastaldi

Geneva, 27 juillet 2015

Place/Date Signature

Principal Co-Investigator:

Dr Sophie Clément

Geneva, 27 juillet 2015

Place/Date Signature

Table of Contents

STUDY SYNOPSIS **5**
STUDY SUMMARY IN LOCAL LANGUAGE **7**
ABBREVIATIONS **8**
STUDY SCHEDULE **9**
1. STUDY ADMINISTRATIVE STRUCTURE **10**
1.1 Sponsor - Principal Investigator 10
1.2 Principal Co-Investigators 10
1.3 Investigational laboratory 10
1.4 Monitoring institution 10
2. ETHICAL AND REGULATORY ASPECTS **11**
2.1 Study registration 11
2.2 Categorisation of study 11
2.3 Competent Authorities (CA) and Ethics Committee (CEC) 11
2.4 Ethical Conduct of the Study 11
2.5 Declaration of interest 11
2.6 Patient Information and Informed Consent 11
2.7 Participant privacy and confidentiality 11
2.8 Early termination of the study 12
2.9 Protocol amendments 12
3. BACKGROUND AND RATIONALE **12**
3.1 Background and Rationale 12
3.2 Translational collaboration and synergism 13
3.3 Medicinal Products administered and Indication 13
3.4 Clinical Evidence to Date 13
3.5 Risks / Benefits 14
3.6 Justification of choice of study population 14
4. STUDY OBJECTIVES **14**
4.1 Overall Objective 14
4.2 Primary Objective 15
4.3 Secondary Objectives 15
5. STUDY OUTCOMES **15**
5.1 Primary Outcome 15
6. STUDY DESIGN **15**
6.1 General study design and justification of design 15
7. STUDY POPULATION **15**
7.1 Eligibility criteria 15
7.2 Compliance with study intervention 16
7.3 Criteria for withdrawal / discontinuation of participants 16
8. EXPERIMENTAL PLAN **16**
8.1 Study duration for subjects and visits schedule 16
8.2 Recruitment, screening and standard medical care 17
8.3 Study treatments 18
8.3.1 Storage Conditions 18

8.4 Intervention visits ... 18
8.5 Data Collection and Follow-up for withdrawn participants ... 18
8.6 Concomitant Interventions and/or treatments ... 19
8.7 Return or Destruction of Study Drug ... 19
9. SAFETY ... 19
9.1 Management of adverse events ... 19
9.1.1 Definition and assessment of (serious) adverse events and other safety related events .. 19
9.1.2 Reporting of serious adverse events (SAE) and other safety related events ... 20
9.1.3 Follow up of (Serious) Adverse Events ... 20
10. STATISTICAL METHODS ... 21
10.1 Determination of Sample Size and Statistical Methods ... 21
10.2 Planned Analyses ... 21
11. QUALITY ASSURANCE AND CONTROL ... 21
11.1 Data handling and record keeping / archiving ... 21
11.1.1 Source Data ... 21
11.1.2 Case Report Forms ... 21
11.1.3 Record keeping / archiving ... 21
11.1.4 Data management ... 21
11.2 Monitoring ... 21
11.3 Confidentiality, Data Protection ... 22
11.4 Audits and Inspections ... 22
11.5 Storage of biological material and related health data ... 22
12. PUBLICATION AND DISSEMINATION POLICY ... 22
13. FUNDING AND SUPPORT ... 22
14. INSURANCE ... 22
15. REFERENCES ... 23
16. ANNEXE 1. PRODUCTS INFORMATION - 09/07/2015 HARVONI -EMEA/H/C/003850 -IB/0008 - RIBAVIRIN – TABLETS (REBETOL®) ... 24

STUDY SYNOPSIS

**Sponsor / Sponsor-Investigator**	Prof. Francesco Negro
**Study Title:**	Hepatitis C virus infection induced insulin resistance: different contribution from liver and extrahepatic sites as inferred by interferon-free antiviral suppression
**Short Title / Study ID:**	Extrahepatic insulin resistance in chronic hepatitis C
**Protocol Version and Date:**	Version number 2 of 27.07.2015
**Trial registration:**	Clinicaltrials.gov
**Study category and Rationale**	Risk category according to LHR : B.<br>Clinical trial with Medicinal Products (MP) authorised in Switzerland for genotype 1 infection but used in this clinical trial on genotype 3 infection (MP already approved in Europe for this application on genotype 3 infection)
**Medicinal Products (MP) Trading Name**	HARVONI®<br>Rebetol®
**Medicinal Products (MP) - Active ingredients:**	ledipasvir and sofosbuvir<br>ribavirin
**Background and Rationale:**	Epidemiological studies have shown that HCV infection induces insulin resistance, which may progress to type 2 diabetes in susceptible individuals. Despite the fact that HCV infects the liver, insulin resistance in these patients appears to originate mostly in extrahepatic tissues, particularly in muscles and adipose tissues.
**Objective(s):**	To assess the relative contribution of hepatic vs extrahepatic tissues to the pathogenesis of insulin resistance in chronic hepatitis C.
**Primary outcome**	Increased (≥10% vs basal) glucose consumption in patients with chronic hepatitis C but without the metabolic syndrome after complete suppression of viral replication induced by 6 weeks of treatment with HARVONI® (Ledipasvir 90 mg/Sofosbuvir 400 mg) and ribavirin, as measured by euglycemic hyperinsulinemic clamp using deuterated glucose, and compared to basal conditions i.e. before antiviral treatment
**Study design:**	20 patients will be enrolled in a single-arm, open-label study. Study subjects will include 10 patients without any feature of the metabolic syndrome, and another 10 with the metabolic syndrome as defined by http://www.idf.org/webdata/docs/MetSyndrome_FINAL.pdf.<br>All patients will receive the same regimen consisting of HARVONI® (Ledipasvir 90 mg/Sofosbuvir 400 mg), one tablet once a day, associated with body weight-dose adjusted, 200 mg-tablets of ribavirin (1,000 mg in two administration in patients <75 Kg of body weight, or 1,200 mg in two administrations for those >75 Kg) for 12 weeks.<br>Insulin resistance will be investigated at baseline (before treatment) and after 6 weeks of treatment.

**Inclusion / Exclusion criteria:**	Inclusion criteria:<br>Histologically confirmed chronic hepatitis C with HCV genotype 3a infection,<br>Adult Caucasian patient males or non-pregnant or non-lactating females, aged 18 to 65 at the time of the screening;<br>Informed Consent as documented by signature;<br>Lack of contraindications to the class of drugs under study, e.g. known hypersensitivity or allergy to class of drugs or the investigational products.<br>Exclusion criteria:<br>Cirrhosis;<br>Excess active alcohol consumption (>30 g/day in males, >20 g/day in females);<br>Active illicit drug use.<br>Coinfection with HIV or HBV;<br>Concomitant medications with clinically significant interactions with the study drugs;<br>Women who are pregnant or breast feeding or who intend to become pregnant during the course of the study;<br>Lack of safe contraception, defined as: female participants of childbearing potential, not using and not willing to continue using a medically reliable method of contraception for the entire study duration, such as oral, injectable, or implantable contraceptives, or intrauterine contraceptive devices, or who are not using any other method considered sufficiently reliable by the investigator in individual cases;<br>Other clinically significant concomitant disease states (e.g., renal failure, hepatic dysfunction, cardiovascular disease, etc.);<br>Known or suspected non-compliance;<br>Inability to follow the procedures of the study, including, but not limited to, language problems, psychological disorders, dementia;<br>Participation in another study with any investigational drug within the 30 days preceding and during the present study;<br>Enrolment of the investigator, his/her family members, employees and other dependent persons.
**Number of Participants with Rationale:**	Number of participants projected for the entire study: 20.
**Study Duration:**	2 years
**Study Duration for patients**	6 months
**Investigator(s):**	Prof. Francesco Negro, Service de Gastroentérologie et d'hépatologie et de Pathologie clinique, Hôpitaux Universitaires de Genève, rue Gabrielle-Perret-Gentil 4, 1211 Genève 14.
**Study Centre(s):**	Hôpitaux Universitaires de Genève, rue Gabrielle-Perret-Gentil 4, 1211 Genève 14.

**Statistical Considerations:**	All results will be expressed as means±SE unless stated otherwise. Comparisons between groups will be performed by multiple way analyses of variance. *Post hoc* comparisons will be made using Fisher's protected least significant difference. Statistical significance will be set at $P < 0.05$. This is a pilot study, and the sample size has been based on feasibility.
**GCP Statement:**	This study will be conducted in compliance with the protocol, the current version of the Declaration of Helsinki, the ICH-GCP as well as all national legal and regulatory requirements.

STUDY SUMMARY IN LOCAL LANGUAGE

Titre de l'étude : La résistance à l'insuline induite par le virus de l'hépatite C: étude de la contribution du foie et des sites extrahépatiques chez des patients atteints d'une hépatite C chronique traités avec une combinaison antivirale sans interféron.

L'infection par le virus de l'hépatite C (VHC) est associée à une résistance à l'insuline, qui peut évoluer vers un diabète de type 2 chez les individus prédisposés. Malgré le fait que le VHC infecte uniquement le foie, plusieurs études ont montré que cette résistance à l'insuline est principalement générée dans des organes extrahépatiques et consiste en une inhibition de la captation et de l'utilisation oxydative du glucose, notamment par le muscle strié. Ceci suggère que les hépatocytes infectés pourraient causer l'insulino-résistance *via* un mécanisme paracrine. Si cette interprétation est correcte, la suppression de la réplication virale – par exemple par des molécules à action antivirale directe – devrait être associée à une amélioration de l'insulino-résistance non seulement hépatique mais aussi périphérique.

Cette étude vise d'une part à évaluer l'influence directe de la charge virale sur la sensibilité à l'insuline du foie, du tissu adipeux et du muscle et, d'autre part, à identifier les acteurs clés impliqués dans la résistance à l'insuline extrahépatique. Nous proposons d'évaluer la résistance à l'insuline chez des patients avant et pendant traitement (c'est-à-dire 6 semaines après le début, lorsqu'on aura atteint une suppression totale de la multiplication virale) avec une combinaison antivirale très puissante, c'est-à-dire HARVONI® (Ledipasvir 90 mg/Sofosbuvir 400 mg) + ribavirine (Rebetol®).

Cette étude pilote, ouverte, à bras unique portera sur 20 patients infectés par le VHC de génotype 3a. On choisira 10 patients sans signe de syndrome métabolique et 10 patients remplissant les critères diagnostiques du syndrome métabolique. Le choix du génotype 3a est justifié par le fait que (i) ce génotype est associé à une stéatose parfois sévère induite directement par la multiplication virale, et on pourra donc évaluer si une corrélation existe entre le degré de stéatose virale et le niveau d'insulino-résistance hépatique et périphérique (chez les patients sans syndrome métabolique), (ii) les médicaments actuellement disponibles en Suisse pour le génotype 3a aboutissent à un taux de guérison sous optimal : cependant, la combinaison proposée – déjà approuvée par les autorités européennes mais pas encore par Swissmedic – est très efficace (100% de guérison), au moins chez une population de patients remplissant les critères d'inclusion et exclusion de l'étude.

Cette combinaison ne serait pourtant pas si efficace chez des patients à un stade avancé de maladie, tels que les cirrhotiques, soulignant la difficulté à traiter ce génotype viral. A deux occasions (avant le traitement et 6 semaines après le début), tous les patients seront soumis à des évaluations très sophistiquées de l'insulino-résistance, en effectuant un clamp hyperinsulinémique euglycémique, une calorimétrie, ainsi que des prélèvements biologiques (sang et tissus adipeux et musculaire).

Des analyses de biologie moléculaire seront faites sur les échantillons de sang et tissu afin d'identifier les médiateurs de l'homéostasie du glucose modifiés lors de la suppression totale de la multiplication virale. Les résultats pourraient aboutir à une meilleure compréhension des mécanismes de la pathogenèse de l'insulino-résistance lors d'une infection par le VHC, permettre de développer des médicaments afin d'améliorer la réponse au traitement de ce génotype difficile à traiter, et servir aussi de modèle pour comprendre la pathogenèse d'autres maladies, telles que le diabète de type 2.

Hôpitaux Universitaires Genève
Rue Gabrielle-Perret-Gentil 4
CH – 1211 Genève 14

ABBREVIATIONS

AE	Adverse Event
CA	Competent Authority (Swissmedic)
CEC	Competent Ethics Committee
CRF	Case Report Form
ClinO	Ordinance on Clinical Trials in Human Research *(in German: KlinV, in French: OClin)*
CTCAE	Common terminology criteria for adverse events
DSUR	Development safety update report
FU	Follow-up
GCP	Good Clinical Practice
IB	Investigator's Brochure
HCV	Hepatitis C Virus
Ho	Null hypothesis
H1	Alternative hypothesis
HFG	Humanforschungsgesetz (Law on human research)
HMG	Heilmittelgesetz
IMP	Investigational Medicinal Product
IST	Investigator Sponsored Trial
IR	Insulin resistance
ISO	International Organisation for Standardisation
ITT	Intention to treat
KlinV	Verordnung über klinische Versuche in der Humanforschung *(in English: ClinO, in French OClin)*
LPTh	Loi sur les produits thérapeutiques
LRH	Loi fédérale relative à la recherche sur l'être humain
OClin	Ordonnance sur les essais cliniques dans le cadre de la recherche sur l'être humain *(in German : KlinV, in English : ClinO)*
PI	Principal Investigator
SDV	Source Data Verification
SOP	Standard Operating Procedure
SPC	Summary of product characteristics
SUSAR	Suspected Unexpected Serious Adverse Reaction
TMF	Trial Master File

Services de Gastroentérologie et
d'hépatologie et de Pathologie clinique

Hôpitaux Universitaires Genève
Rue Gabrielle-Perret-Gentil 4
CH – 1211 Genève 14

STUDY SCHEDULE

Study Periods	Screening	Baseline		Treatment						Follow-up	
Visit	1	2		3	4	5	6		7	8	9
	SC*	SC*	EXP**	SC*	SC*	SC*	SC*	EXP**	SC*	SC*	SC*
Time (days)	-120 → -30d	-30 → -1d		0	14 ± 2d	28 ± 2d	42 ± 4d		84 ± 7d	112 ± 7d	168 ± 7d
Informed Consent	x										
Demographics	x										
Medical History	x										
Inclusion/Exclusion Criteria	x										
Physical Examination	x	x		x							x
Vital Signs	x	x		x	x	x	x	x	x	x	x
Laboratory Tests	x	x					x		x	x	x
Pregnancy Test	x	x		x	x	x	x		x	x	x
Serum HCV RNA	x	x		x	x	x	x		x	x	x
Investigations at CRC			x					x			
Scan (Radiology)				x					x		
Concomitant Therapy	x	x		x	x	x	x		x		
Adverse Events EXP			x					x			
Adverse Events Treatment				x	x	x	x		x	x	x
End of the study											x

*SC = standard care procedures

**EXP = experimental procedures

1. STUDY ADMINISTRATIVE STRUCTURE

1.1 Sponsor - Principal Investigator

Prof Francesco Negro, MD, Sponsor and Principal Investigator

1.2 Principal Co-Investigators

Dr Giacomo Gastaldi, MD, Principal Co-Investigator

Dr Sophie Clément, PhD, Principal Co-Investigator

1.3 Investigational laboratory

Prof Luc Tappy

1.4 Monitoring institution

The UIC-CRC Centre de Recherche Clinique des Hopitaux Universitaires de Genève will be in charge of the monitoring for the clinical trial.

Hôpitaux Universitaires Genève
Rue Gabrielle-Perret-Gentil 4
CH – 1211 Genève 14

2. ETHICAL AND REGULATORY ASPECTS

2.1 Study registration

The study will be registered at the Clinicaltrials.gov website and at the Swiss Federal Complementary Database (Portal).

2.2 Categorisation of study

Risk category according to LHR: B.

Clinical trial with Medicinal Products (MP) (HARVONI® + Ribavirin) authorised in Switzerland for genotype 1 infection but used in this clinical trial on genotype 3 infection (MP already approved in Europe for this application on genotype 3 infection).

2.3 Competent Authorities (CA) and Ethics Committee (CEC)

The Sponsor ensures that approval from an appropriately constituted Competent Ethics Committee (CEC) and Swissmedic is sought for the clinical study. No changes are made to the protocol without prior Sponsor, Swissmedic and CEC approval. Premature study end or interruption of the study is reported within 15 days. The regular end of the study is reported to the CEC within 90 days, the final study report shall be submitted within one year after study end.

2.4 Ethical Conduct of the Study

The study will be carried out in accordance to the protocol and with principles enunciated in the current version of the Declaration of Helsinki, the guidelines of Good Clinical Practice (GCP) issued by ICH, the Swiss Law and Swiss regulatory authority's requirements. The CEC and regulatory authorities will receive annual safety and interim reports and be informed about study stop/end in agreement with local requirements.

2.5 Declaration of interest

The Sponsor is member of the Gilead Sciences Advisory Board.

2.6 Patient Information and Informed Consent

The investigators will explain to each subject the nature of the study, its purpose, the procedures involved, the expected duration, the potential risks and benefits and any discomfort it may entail. Each subject will be informed that the participation in the study is voluntary and that he/she may withdraw from the study at any time and that withdrawal of consent will not affect his/her subsequent medical assistance and treatment.

The subject must be informed that his/her medical records may be examined by authorised individuals other than their treating physician.

All subjects for the study will be provided a participant information sheet and a consent form describing the study and providing sufficient information for participant to make an informed decision about their participation in the study. The patient information sheet and the consent form have been submitted to the CEC to be reviewed and approved. The formal consent of a participant, using the approved consent form, must be obtained before the subject is submitted to any study procedure.

The subject should read and consider the statement before signing and dating the informed consent form, and should be given a copy of the signed document. The consent form must also be signed and dated by the investigator (or his designee) and it will be retained as part of the study records.

2.7 Participant privacy and confidentiality

The investigator affirms and upholds the principle of the participant's right to privacy and that they shall comply with applicable privacy laws. Especially, anonymity of the participants shall be guaranteed when presenting the data at scientific meetings or publishing them in scientific journals.

Individual subject medical information obtained as a result of this study is considered confidential and disclosure to third parties is prohibited. Subject confidentiality will be further ensured by utilising subject identification code numbers to correspond to treatment data in the computer files.

For data verification purposes, authorised representatives of the Sponsor (-Investigator), a competent authority (e.g. Swissmedic), or an ethics committee may require direct access to parts of the medical records relevant to the study, including participants' medical history.

2.8 Early termination of the study

The Sponsor-Investigator may terminate the study prematurely according to the following circumstances:

- Any ethical concerns;
- Insufficient participant recruitment within two years from start;
- When the safety of the participants is doubtful or at risk, respectively;
- Alterations in accepted clinical practice that make the continuation of this clinical trial unwise.

2.9 Protocol amendments

Substantial amendments are only implemented after approval of the CEC and CA respectively.

Under emergency circumstances, deviations from the protocol to protect the rights, safety and well-being of human subjects may proceed without prior approval of the sponsor and the CEC/CA. Such deviations shall be documented and reported to the sponsor and the CEC/CA as soon as possible.

All Non-substantial amendments are communicated to the CA as soon as possible if applicable and to the CEC within the Annual Safety Report (ASR).

3. BACKGROUND AND RATIONALE

3.1 Background and Rationale

The hepatitis C virus (HCV) is a major health issue worldwide. The global health burden related to HCV infection encompasses both hepatic and extrahepatic morbidity and mortality. In fact, in addition of being a leading cause of end-stage liver disorders (decompensated cirrhosis and hepatocellular carcinoma), HCV is associated with a wide array of extrahepatic disorders that can be fatal (insulin resistance, type 2 diabetes and some cardiovascular complications such as atherosclerosis and ischemic stroke) [1]. Several data show that antiviral therapy reduces the post-treatment incidence of glucose metabolic disturbances, including type 2 diabetes, and that treatment of chronic hepatitis C patients with diabetes may reduce the incidence of its complications, notably end-stage renal disease and stroke [2].

The pathogenesis of type 2 diabetes associated with HCV infection involves insulin resistance. Several studies have shown that patients with chronic hepatitis C have C-peptide levels and insulin resistance scores that are significantly increased compared to individuals with chronic hepatitis B matched for other usual risk factors for metabolic alterations, including liver fibrosis scores [3]. Since HCV infects primarily hepatocytes, it is intuitive to imply a direct interaction between HCV products and the insulin signalling cascade inside the infected cells. Indeed, several post-receptor mechanisms have been reported both in experimental models and in human livers, accounting for the increased insulin resistance (reviewed in [4]). However, recent work has pinpointed a more indirect mechanism involving the secretion of hitherto unidentified substances by infected hepatocytes and capable of altering metabolically sensitive tissues, such as the adipose and striated muscles tissues.

In a first study [5], 14 patients with chronic hepatitis C with or without steatosis but lacking any feature of the metabolic syndrome were subjected to euglycemic hyperinsulinemic clamp coupled with infusion of deuterated glucose and glycerol and indirect calorimetry. Results were compared with those obtained in healthy controls and showed a reduction of glucose oxidative disposal in hepatitis C patients, coupled with a slightly increased endogenous glucose production that was less suppressed during the clamp procedure. These data showed the presence of a significant extrahepatic component of insulin resistance, essentially located in skeletal muscle. The authors also postulated an enhanced hepatic expression of inflammatory cytokines/mediators potentially involved in the defective glucose regulation via endocrine mechanisms.

A second study using a similar approach confirmed that the hepatic glucose production and non-esterified free fatty acid suppression with insulin observed in chronic hepatitis C were comparable to controls, suggesting that the HCV-induced insulin resistance is confined to muscle and is not involving adipose tissue. The authors concluded saying that chronic hepatitis C is an infective/inflammatory model

of insulin resistance, predominantly originating in muscle, and independent of steatosis [6].

These results have introduced the important concept that an infectious agent like HCV is capable of influencing metabolic alterations in uninfected tissues, although the nature of the endocrine mediators is unknown. The missing piece of evidence should come from the reversal of these effects via the effective therapy with direct antivirals. The existing data show that during therapy with interferon (IFN) alpha-containing regimens there may be a reduction in the insulin resistance scores [7]. However, IFN alpha is per se capable of altering the insulin signalling transduction pathway via tyrosine phosphorylation of the insulin receptor substrate-1 [8]. The recent arrival into the market of direct-acting antivirals (DAA) to be administered in IFN-free regimens will allow solving this issue, at least in some HCV genotype infections [9].

In this pilot study, we plan to treat patients with chronic hepatitis C due to HCV genotype 3 infection using an IFN-free regimen consisting in the administration of ribavirin and HARVONI® (sofosbuvir/ledipasvir - a combination of a nucleotide RNA polymerase inhibitor with a NS5A inhibitor - that has been approved by Swissmedic in January 2015 for the treatment of HCV genotype 1 infection). Patients will undergo a thorough study with euglycemic hyperinsulinemic clamp, using tracers, and indirect calorimetry to assess whether the virtual universal viral suppression reported with this regimen [10] will be capable of reversing the glucose metabolic alterations induced by HCV in both the liver and extrahepatic compartments. Adipose and muscle tissue biopsies will be performed to assess some specific molecular changes induced by HCV.

3.2 Translational collaboration and synergism

The research effort described in this proposal will be orchestrated between two laboratories focusing on the effect of HCV viral load on insulin resistance in patients. The sharing of key expertise and research tools should benefit each group to speed up their scientific advancements and to decrease experimental costs.

Indeed both groups will work in close synergy because of their complementary skills: the group of Professor Negro has the expertise and tools for the investigation and interpretation of the mechanisms of insulin resistance. In addition, Prof Negro has access to a HCV-infected cohort of ~600 patients actively followed at the Division of Gastroenterology and Hepatology of the University Hospitals of Geneva. The group of Dr Gastaldi will provide the experimental protocols and skills for euglycemic hyperinsulinemic clamp and indirect calorimetry experiments. In addition, Dr Clément, who is a senior scientist in Prof Negro's laboratory, has a long-standing experience and has extensively published in the fields of molecular biology and virology.

3.3 Medicinal Products administered and Indication

HARVONI® contains the prescription medicines ledipasvir and sofosbuvir.

Sofosbuvir is a uridine analogue, nucleotide inhibitor of the HCV NS5B RNA-dependent RNA polymerase. It is currently marketed in Switzerland as oral 400 mg tablet under the trade name SOLVADI®. Ledipasvir is a HCV NS5A inhibitor marketed in Switzerland in oral tablets containing a fixed dose combination of 90 mg of ledipasvir. The combination of 90 mg of ledipasvir and 400 mg of sofosbuvir is commercialized under the trade name of HARVONI®. Ledipasvir is not available as a stand-alone drug.

Ribavirin is an oral compound used as antiviral against several infections: its mechanism of action is multifaceted. In Switzerland, it is marketed as 200 mg oral tablets under the trade names Rebetol®. It is prescribed in combination with other drugs active against HCV. Currently, the only drugs that can be combined with ribavirin, as per Swiss label, are interferon alpha-2a and -2b, including their pegylated forms, all marketed HCV protease inhibitors (telaprevir, boceprevir and simeprevir) and sofosbuvir. In other countries, including the US and the EU, it can be also combined with HARVONI®. Further details of efficacy and safety can be found in the "European public assessment report (EPAR)" and in the HARVONI® Investigator's Brochure (Products information can be also found in the annex 1 of this protocol).

3.4 Clinical Evidence to Date

While already indicated in Europe for genotype 1 and 3, this combination of drug has been approved so far by Swissmedic for genotype 1 only. It is noteworthy that this combination has been admitted for

genotypes 3 and 4 in Europe, also in combination with ribavirin.

3.5 Dose Rationale

All patients will receive a regimen consisting of HARVONI®, one tablet once a day, associated with body weight-dose adjusted, 200 mg-tablets of ribavirin (1,000 mg in two administration in patients <75 Kg, or 1,200 mg in two administrations for those >75 Kg) for 12 weeks, as reported in the EMA indication.

3.6 Risks / Benefits

Patients will receive the most effective and better tolerated treatment available for the infection with the genotype 3 of HCV without cirrhosis. The previous results of the ELECTRON-2 study, an open-label, phase 2 study enrolling 26 treatment-naïve chronic hepatitis C patients with genotype 3 infection [10] have shown a 100% efficacy using this same fixed-dose combination of ledipasvir-sofosbuvir ± ribavirin in this kind of patients' population. In addition, patients enrolled in the study will undergo a thorough evaluation of glucose metabolism, which may prove useful to assess their cardiovascular risk and provide guidance as to their lifestyle.

The administration of HARVONI® may be associated with various SE, although it is in general well tolerated. Very frequent SE (>10% of cases) include fatigue and headache. Frequent SE (1-10% of cases) include nausea, insomnia, diarrhea.

The administration of ribavirin may induce side effects (SE). Very frequent SE include a reduction of hemoglobin, nausea, headache, hyperbilirubinemia, fatigue. These SE can be controlled by ribavirin dose adjustments. Frequent SE include cough, dyspnea, reduced level of attention, dyspepsia, constipation, abdominal discomfort, itching, myalgia. Ribavirin is potentially teratogenic in humans, and all precautions will be taken to avoid unwanted pregnancies during the study period in all participants and their spouses.

Additional details about SE occurring to a less frequency in patients receiving HARVONI® and ribavirin can be found in the annex 1 of this protocol and at the website http://www.compendium.ch/

There are no competing trials at the study site that may interfere with patients' recruitment.

3.7 Justification of choice of study population

Chronic hepatitis C patients with genotype 3 infection are characterised by a distinct disease phenotype, including a moderate to severe liver steatosis, an accelerated liver fibrosis progression rate, an increased risk of the development of hepatocellular carcinoma and a reduced virological response to IFN-free regimens [11]. Thus, patients with genotype 3 have a worse clinical outcome and, in addition, at variance with those with the most frequently encountered genotype 1, have less curative options. Insulin resistance is a known liver disease modifier capable of increasing the rate of fibrosis progression and the risk of hepatocellular carcinoma development: thus, we think that unraveling the pathogenesis of insulin resistance in these patients may contribute to a better control of the morbidity associated with genotype 3, and possibly to improve treatment options.

4. STUDY OBJECTIVES

4.1 Overall Objective

To study the mechanisms underlying the insulin resistance found in chronic hepatitis C.

HCV induces insulin resistance, which in turn impacts liver disease progression. Despite the fact that HCV infects primarily the liver, most HCV-induced insulin resistance originates at extrahepatic uninfected sites, especially the skeletal muscle. However, the molecular mechanisms by which HCV induces extra-hepatic insulin resistance are unknown. Research efforts described in this proposal should shed light on the impact of HCV infection on hepatic and skeletal glucose disposal in insulin resistant patients. Indeed, the reversal of these effects *via* the effective therapy with direct new antiviral interferon-free treatments should provide additional mechanistic information. This study should draw for the first time a comprehensive picture of the situation of these patients, as we aim to collect data ranging from the physiology to the molecular level. Since the spectrum of diseases associated with HCV infection is similar in some aspects to metabolic syndrome, our studies will likely provide also new knowledge and concepts to understand metabolic disorders in general. Therefore, such findings may

also have the potential to provide on new therapeutic tools for the management of diabetes.

4.2 Primary Objective

The primary aim of this study is to assess the relative contribution of hepatic vs extrahepatic tissues to the pathogenesis of insulin resistance in chronic hepatitis C, with specific emphasis on patients infected with the genotype 3 of HCV, characterized, as detailed above, by a distinctly aggressive phenotype. This goal will be reached by measuring the level of hepatic and extrahepatic insulin resistance in 20 patients at baseline and after complete suppression of the viral replication by administering a potent antiviral combination. Patients will be divided in two groups: 10 with criteria fulfilling the diagnosis of metabolic syndrome, and 10 without any such criteria, in whom insulin resistance (if any is present) will be deemed as virus-induced. This virus-induced feature should in principle be suppressed by an effective antiviral therapy, while no (or a very reduced effect) will be observed in patients with the metabolic syndrome).

4.3 Secondary Objectives

A secondary objective will be to identify markers associated with the change in virus-induced insulin resistance, that may provide the rationale for translational studies aimed at identifying the mechanisms underlying the insulin resistance induced by HCV.

5. STUDY OUTCOMES

5.1 Primary Outcome

The primary endpoint of the study is an increased (≥10% vs basal) glucose consumption in patients with chronic hepatitis C but without the metabolic syndrome after complete suppression of viral replication induced by 6 weeks of treatment with HARVONI® and ribavirin, as measured by euglycemic hyperinsulinemic clamp using deuterated glucose, and compared to basal conditions i.e. before antiviral treatment.

6. STUDY DESIGN

6.1 General study design and justification of design

We will include 20 patients in an open-label, single arm pilot clinical trial. The study population will consist of 10 patients without metabolic syndrome, and 10 patients with the metabolic syndrome, as per the most recent definition (http://www.idf.org/webdata/docs/MetSyndrome_FINAL.pdf). This will allow to identify virus-induced metabolic effects by comparing outcome measures at baseline vs those observed after 6 weeks of complete suppression of viral replication. In patients with genotype 3 and the metabolic syndrome, we expect to observe reduced changes, since most of the metabolic outcome measures will be host-induced.

7. STUDY POPULATION

7.1 Eligibility criteria

Participants fulfilling all of the following inclusion criteria are eligible for the study:

- Histologically confirmed chronic hepatitis C with HCV genotype 3a infection,
- Adult Caucasian patient males or non-pregnant or non-lactating females, aged 18 to 65 at the time of the screening;
- Informed Consent as documented by signature;
- Lack of contraindications to the class of drugs under study, e.g. known hypersensitivity or allergy to class of drugs or the investigational products.

The presence of any one of the following <u>exclusion criteria</u> will lead to exclusion of the participant:

- Cirrhosis;
- Excess active alcohol consumption (>30 g/day in males, >20 g/day in females);
- Active illicit drug use.
- Coinfection with HIV or HBV;
- Concomitant medications with clinically significant interactions with the study drugs;
- Women who are pregnant or breast feeding or who intend to become pregnant during the course of the study;
- Lack of safe contraception, defined as: female participants of childbearing potential, not using and not willing to continue using a medically reliable method of contraception for the entire study duration, such as oral, injectable, or implantable contraceptives, or intrauterine contraceptive devices, or who are not using any other method considered sufficiently reliable by the investigator in individual cases;
- Other clinically significant concomitant disease states (e.g., renal failure, hepatic dysfunction, cardiovascular disease, etc.);
- Known or suspected non-compliance;
- Inability to follow the procedures of the study, including, but not limited to, language problems, psychological disorders, dementia;
- Participation in another study with any investigational drug within the 30 days preceding and during the present study;
- Enrolment of the investigator, his/her family members, employees and other dependent persons.

7.2 Compliance with study intervention

Patients will be instructed about the importance of taking the study medications on a regular basis (i.e. in order to optimize the chances of efficacy), and about the risks of failing to do so, with particular emphasis on the selection of resistant-associated viral variants. In addition, patients will be asked to return the empty boxes to allow precise counting of administered pills.

7.3 Criteria for withdrawal / discontinuation of participants

The reasons for withdrawal include: non-compliance to the treatment or the study protocol procedure; clinically significant SE due to the treatment or to the metabolic investigation; disease progression; personal reasons (including – but not limited to – death of a loved one, relocation, loss of income, marriage/divorce or change in job), lost to follow-up, pregnancy during the treatment phase of the study or the six months following the last dosing of ribavirin. In case of withdrawal, patients will undergo – if willing or capable to do so – medical FU as indicated.

8. EXPERIMENTAL PLAN

8.1 Study duration for subjects and visits schedule

Subjects will be enrolled in the study for a period of 6 months.

Study Periods	Screening	Baseline		Treatment						Follow-up	
Visit	1	2		3	4	5	6		7	8	9
	SC*	SC*	EXP**	SC*	SC*	SC*	SC*	EXP**	SC*	SC*	SC*

Time (days)	-120 → -30d	-30 → -1d		0	14 ± 2d	28 ± 2d	42 ± 4d		84 ± 7d	112 ± 7d	168 ± 7d
Informed Consent	x										
Demographics	x										
Medical History	x										
Inclusion/Exclusion Criteria	x										
Physical Examination	x	x		x							x
Vital Signs	x	x		x	x	x	x	x	x	x	x
Laboratory Tests	x	x					x		x	x	x
Pregnancy Test	x	x		x	x	x	x		x	x	x
Serum HCV RNA	x	x		x	x	x	x		x	x	x
Investigations at CRC			x					x			
Scan (Radiology)				x					x		
Concomitant Therapy	x	x		x	x	x	x		x		
Adverse Events EXP			x					x			
Adverse Events Treatment				x	x	x	x		x	x	x
End of the study											x

*SC = standard care procedures

**EXP = experimental procedures

8.2 Recruitment, screening and standard medical care

The selection of patients for the study will be performed during a routine consultation. Before any study-specific activities/procedure, the appropriate written informed consent must be obtained.

Before treatment start, we will record the patients' body weight and height.

Blood samples will be taken for HCV RNA determination (by quantitative real-time RT-PCR), glucose and insulin measurement (HOMA-IR score calculation), and usual tests as per good clinical practice before and during antiviral therapy (liver enzymes, hematogram, and creatininemia)

Whatever the type of antiviral treatment administered, our current practice is to provide all monitoring visits required. These visits (standard care medical visits and procedures) are generally performed at 2 weeks (14 days) after the introduction of treatment, at 4 weeks (28 days), at 6 weeks (42 days) and at the end of treatment, i.e. at 12 weeks (84 days), and then at 4 (112 days) and 12 weeks (168 days) after the end of treatment in order to ascertain cure. We will use the same planning for patients enrolled in the study. Patients will undergo all routine examinations and we will use the data collected for the study. During this kind of visits, patients are requested only to allow the collection of an extra tube of blood (~10 mL) for the study, but the material and the procedures associated with this are entirely free of charge.

All patients will receive a regimen consisting of HARVONI®, one tablet once a day, associated with body weight-dose adjusted, 200 mg-tablets of ribavirin (1,000 mg in two administration in patients <75 Kg, or 1,200 mg in two administrations for those >75 Kg) for 12 weeks.

Treatment adherence will be monitored by counting the remaining pills at the FU visits. Response to the treatment will be evaluated by measuring the viral titer before, during (after 2 and 4 weeks) and at the end of the treatment.

8.3 Study treatments

HARVONI® will be provided by Gilead and Ribavirin (Rebetol®) will be purchased by the Pharmacy of HUG. Both products are commercially available medicinal products and a considerable amount of information such as batch number, expiry date and storage conditions is already included on the normal commercial label. Upon reception, the Pharmacy of HUG will take care of adding a special label for both drugs according to the indications of Swissmedic and with the following elements: Trial number or Trial ID; Trial subject number / Patient ID; Name of the sponsor, investigator, and contact details.

The batch number will be noted in the CRF.

8.3.1 Storage Conditions

All study medications will be stored in the original packing at a temperature comprised between 8° and 20° C, protected from light.

8.4 Intervention visits

To assess the relative contribution of hepatic *vs.* extrahepatic tissues to the pathogenesis of insulin resistance in chronic hepatitis C, the experimental intervention visits will be performed at baseline (within 30 days before the introduction of the treatment) and after six weeks (42 days) of treatment (i.e. at a time when viral replication will have been completely suppressed). For both visits, patients will be admitted to the hospital in the morning, after an overnight fasting period (10–12 h) and will undergo the following set of tests:

i. Insulin resistance will be measured using the euglycemic hyperinsulinemic clamp with non-radioactive tracers (deuterium-labeled glucose and glycerol) technique as previously described [12]. Isotopes are produced by Cambridge Isotope Laboratories, Innerberg, Switzerland. Sterile pathogen-free solutions will be made by the University Hospital Pharmacy, CHUV, Lausanne, Switzerland following the attached protocols (Annex 2). Throughout the experiments, respiratory gas exchanges will be monitored by open-circuit indirect calorimetry as described [13]. Plasma glucose will be determined by a biosensor analyse (enzymatic method), using a Yellow Spring Instrument YSI 2900 (YSI instrument). Plasma free fatty acid concentrations will be determined by a colorimetric assay kit (Wako). Plasma lactate concentrations will be measured enzymatically. Plasma insulin concentrations will be measured by radioimmunoassay (kit from Biodata Guidonia-Montecelio). Urinary urea concentration will be analysed enzymatically. Plasma [6,6-2H2]glucose will be measured by gas chromatography-mass spectrometry, as described [14]. The rates of glucose appearance (Ra) and disappearance (Rd) will be calculated from plasma [6,6-2 H2]glucose enrichment using hot infusate equations [15].

ii. Regional soft tissue composition will be assessed at the 20% femur (thigh) and liver using unenhanced computed tomography (CT). Edge- detection and threshold techniques will be used to separate tissues (i.e., adipose, muscle, and bone) based on attenuation characteristics, which are directly related to tissue composition and density [16, 17]. To measure hepatic steatosis three methods will be evaluated: the measurement of hepatic attenuation (CT L), the ratio of hepatic attenuation to splenic attenuation (CT L/S), and the difference between the hepatic and splenic attenuation (CT L-S) [18]. The total dose length product (DLP) will be about 60 mGy.cm, which corresponds to 0.6 mSv (very small dose when compared to the average annual radiation exposure in Switzerland of 5.6 mSv).

iii. Muscle and adipose tissue biopsies will be obtained using a Magnum® Reusable Core Biopsy Instrument (Bard biopsy system).

The costs of all the procedures detailed in the above paragraph 8.4 are entirely covered by the study.

8.5 Data Collection and Follow-up for withdrawn participants

In case a patient is withdrawn from the study before any of the euglycemic hyperinsulinemic clamp assessment, he/she will be replaced to allow for the predetermined number of enrolments to be completed. No additional study assessments are foreseen for those patients who are lost to FU after

the clamp determinations.

8.6 Concomitant Interventions and/or treatments

Patients cannot undergo medical treatment that may modify the antiviral efficacy of HARVONI® and/or ribavirin during the study period. In addition, safety issues may arise from concomitant medications taken during the study period. The potential drug-drug interactions that may affect the safety and antiviral efficacy or HARVONI® and/or ribavirin are listed and discussed in online databases (e.g. hep-druginteractions.com) or in the Swiss Compendium. In addition, patients are not allowed to undergo medical and/or elective surgical treatments during the six week study period that may affect the metabolic homeostasis thus altering the study endpoints.

8.7 Return or Destruction of Study Drug

Any unused study medications will be destroyed.

9. SAFETY

9.1 Management of adverse events

9.1.1 Definition and assessment of (serious) adverse events and other safety related events

An **Adverse Event (AE)** is any untoward medical occurrence in a patient or a clinical investigation participant administered a pharmaceutical product and which does not necessarily have a causal relationship with the study procedure. An AE can therefore be any unfavourable and unintended sign (including an abnormal laboratory finding), symptom, or disease temporally associated with the use of a medicinal (investigational) product, whether or not related to the medicinal (investigational) product.

Any AE will be assessed and documented. It will be assigned one of three levels: slight, moderate, and severe.

A **Serious Adverse Event (SAE)** is classified as any untoward medical occurrence that:

- results in death,
- is life-threatening,
- requires in-patient hospitalization or prolongation of existing hospitalisation,
- results in persistent or significant disability/incapacity, or
- is a congenital anomaly/birth defect.

In addition, important medical events that may not be immediately life-threatening or not result in death, or not require hospitalisation, but may require intervention to prevent one of the other outcomes listed above should also usually be considered serious.

Assessment of Causality

Both Sponsor-investigator and Co-Investigators make a causality assessment of the event to the study drug, based on the criteria listed in the ICH E2A guidelines:

Relationship		Description
4	Definitely	Temporal relationship<br>Improvement after dechallenge*<br>Recurrence after rechallenge<br>(or other proof of drug cause)
3	Probably	Temporal relationship<br>Improvement after dechallenge<br>No other cause evident
2	Possibly	Temporal relationship<br>Other cause possible
1	Unlikely	Any assessable reaction that does not fulfil the above conditions

0	Not related	Causal relationship can be ruled out

Unexpected Adverse Drug Reaction

An "unexpected" adverse drug reaction is an adverse reaction, the nature or severity of which is not consistent with the applicable product information.

Suspected Unexpected Serious Adverse Reactions (SUSARs)

The Sponsor-Investigator evaluates any SAE that has been reported regarding seriousness, causality and expectedness. If the event is related to the investigational product and is both serious and unexpected, it is classified as a SUSAR.

Assessment of Severity

The degree of causality between the administration of the pharmaceutical products and the adverse reaction is evaluated as definitely, probably, possibly, unlikely and not related.

9.1.2 Reporting of serious adverse events (SAE) and other safety related events

The investigators will systematically search for AEs (history taking, clinical examination, laboratory values etc.). AEs will be documented in the source documents as well as in the CRF. Severity and causality linked to the assessed product will be assessed immediately.

Reporting of SAEs

SAEs resulting in death are reported to the local Ethics Committee (via local Investigator) within 7 days.

Any SUSAR will be reported to the local Ethics Committee (local event via local Investigator) and to Swissmedic (via Sponsor-Investigator) within 7 days, if the event is fatal, or within 15 days (all other events).

SAEs will be reported annually as an annual safety report.

Reporting of Safety Signals

All suspected new risks and relevant new aspects of known adverse reactions that require safety-related measures, i.e. so called safety signals, report the safety signals within 7 days to the local Ethics Committee and to Swissmedic.

Reporting and Handling of Pregnancies

Pregnant participants must immediately be withdrawn from the clinical study. Any pregnancy during the treatment phase of the study and within 30 days after discontinuation of study medication will be reported to the Sponsor-Investigator within 24 hours. The course and outcome of the pregnancy should be followed up carefully, and any abnormal outcome regarding the mother or the child should be documented and reported.

Expected Adverse Drug Reaction

The administration of HARVONI® may be associated with various SE, although it is in general well tolerated. Very frequent SE (>10% of cases) include fatigue and headache. Frequent SE (1-10% of cases) include nausea, insomnia, diarrhea.

The administration of ribavirin may induce side effects (SE). Very frequent SE include a reduction of hemoglobin, nausea, headache, hyperbilirubinemia, fatigue. These SE can be controlled by ribavirin dose adjustments. Frequent SE include cough, dyspnea, reduced level of attention, dyspepsia, constipation, abdominal discomfort, itching, myalgia. Ribavirin is potentially teratogenic in humans, and all precautions will be taken to avoid unwanted pregnancies during the study period in all participants and their spouses.

Additional details about SE occurring to a less frequency in patients receiving HARVONI® and ribavirin can be found at the website http://www.compendium.ch/ and at the annexe 1 of this protocol.

9.1.3 Follow up of (Serious) Adverse Events

Principal investigator and/or Co-Investigators will be available 24/24h, 7/7days to answer any question

of study participants linked to study procedures or concerning AEs. This physician will be available after contacting the gastroenterologist on call at the numbers 079 55 34520 (day) or 074 055 02 87 (night). Any AE will be carefully followed-up until the end of the study.

10. STATISTICAL METHODS

10.1 Determination of Sample Size and Statistical Methods

This is a pilot study, and the sample size has been based on feasibility.

All results will be expressed as means±SE unless stated otherwise. Comparisons between groups will be performed by multiple way analyses of variance. *Post hoc* comparisons will be made using Fisher's protected least significant difference. Statistical significance will be set at $P < 0.05$.

10.2 Planned Analyses

Comparisons between groups will be performed by multiple way analyses of variance. *Post hoc* comparisons will be made using Fisher's protected least significant difference.

11. QUALITY ASSURANCE AND CONTROL

The Sponsor is responsible for implementing and maintaining quality assurance and quality control systems with written SOPs and Working Instructions, at all sites in case of multicentre studies. The PI is responsible for proper training of all involved study personnel.

11.1 Data handling and record keeping / archiving

11.1.1 Source Data

Source data collected during Standard Care Visits used for the study will be collected in the medical records of the patients. All data will be collected and stored in an individual folder per patient, which will contain CRF, SAEs, AEs forms, Informed Consent Forms, drug information provided by the pharmacy.

These are original reports, clinical data, laboratory records, etc. Source data will be made available by the primary investigator in case of monitoring, audit or inspection to the entitled persons.

11.1.2 Case Report Forms

The CRF will be filled the day of each investigation.

The following data will be collected: demographic data, visit dates, concomitant medication, biochemical and viral data (extracted from the clinical file), results of the metabolic analyses, adverse events. All source data and CRFs will be stored securely.

11.1.3 Record keeping / archiving

All study data must be archived for a minimum of 10 years after study termination or premature termination of the clinical trial.

11.1.4 Data management

The Principal Investigator will have the responsibility for supervising all data that accrue from the study by designated persons. Throughout regular data collection, clinical data will be reported on CRFs. This includes safety data, safety laboratory data and outcome data.

11.2 Monitoring

Throughout the study, a monitoring will be realised by the Clinical Research Unit of Geneva University Hospitals to verify the good application of the study protocol and the concordance between the source data and the CRF, the modifications of the CRF and the collection of the adverse effects.

11.3 Confidentiality, Data Protection

The list with the names and contact data of study participants and the corresponding study code will be protected and made accessible only to the study investigators, the ethical committee (EC) and regulatory authorities in accordance with the applicable regulatory requirements. All study documents are attributable to a subject only by a triple digit code. The EC, and regulatory authorities will be granted direct access to all the subject's records for verification of clinical trial procedures and/or data, without violating the confidentiality of the subject, to the extent permitted by the applicable laws and regulations and that, by signing a written informed consent form, the subject is authorizing such access.

11.4 Audits and Inspections

In case of an audit or inspection by the regulatory authorities, the investigator will allow the authorized persons to:
- visit the study local, installations and used material;
- meet all the persons involved from near and far in the study;
- have a direct access at the study documents and source data;
- see all the documents related to the study.

11.5 Storage of biological material and related health data

Clinical samples will be stored at HUG for 15 years and then destroyed.

12. PUBLICATION AND DISSEMINATION POLICY

Data obtained from this study will be subject to publication in a peer reviewed journal. After obtaining the agreement by the Ethical Committee and Swissmedic, this clinical trial will be registered on the international register of clinical trials (www.clinicaltrial.gov), in accordance with the requirements of the international committee of editors of medical reviews.

13. FUNDING AND SUPPORT

The study is supported by a grant from Gilead Sciences, which will cover all investigation-related procedures and the study nurses salary. Private research funds from the Sponsor-Principal Investigator will cover costs related to research.

Gilead Sciences will also provide Harvoni® to all enrolled patients. Ribavirin costs will be supported by study grant.

14. INSURANCE

The promoter is a member of Geneva University Hospitals so the insurance will be covered by Zurich Insurance. A contract with the insurance will be signed to cover the damages which could arise in this particular study.

15. REFERENCES

1. Negro F. Facts and fictions of HCV and comorbidities: steatosis, diabetes mellitus and cardiovascular diseases. J Hepatol 2014;61(suppl 1):S69-S78.
2. Hsu YC, Ho HJ, Huang YT, Wang HH, Wu MS, Lin JT, Wu CY. Association between antiviral treatment and extrahepatic outcomes in patients with hepatitis C virus infection. Gut 2015;64:495-503.
3. Mason AL, Lau JY, Hoang N, Qian K, Alexander GJ, Xu L, et al. Association of diabetes mellitus and chronic hepatitis C virus infection. Hepatology 1999;29:328-33.
4. Kaddai V, Negro F. Current understanding of insulin resistance in hepatitis C (review). Expert Rev Gastroenterol 2011;5:503-16.
5. Vanni E, Abate ML, Gentilcore E, Hickman I, Gambino R, Cassader M, et al. Sites and mechanisms of insulin resistance in nonobese, nondiabetic patients with chronic hepatitis C. Hepatology 2009;50:697-706.
6. Milner KL, van der Poorten D, Trenell M, Jenkins AB, Xu A, Smythe G, et al. Chronic hepatitis C is associated with peripheral rather than hepatic insulin resistance. Gastroenterology 2010;138:932-941 e931-933.
7. Kawaguchi T, Ide T, Taniguchi E, Hirano E, Itou M, Sumie S, et al. Clearance of HCV improves insulin resistance, beta-cell function, and hepatic expression of insulin receptor substrate 1 and 2. Am J Gastroenterol 2007;102:570-6.
8. Platanias LC, Uddin S, Yetter A, Sun XJ, White MF. The type I interferon receptor mediates tyrosine phosphorylation of insulin receptor substrate 2. J Biol Chem 1996;271:278-82.
9. Pawlotsky JM, Feld JJ, Zeuzem S, Hoofnagle JH. From non-A, non-B hepatitis to hepatitis C virus cure. J Hepatol 2015;62(1 Suppl):S87-99.
10. Gane EJ, Hyland RH, An D, Pang PS, Symonds WT, McHutchison JG, Stedman CA. Ledipasvir/sofosbuvir fixed-dose combination is safe and effective in difficult-to-treat populations including GT 3 patients, decompensated GT 1 patients, and GT 1 patients with prior sofosbuvir experience. Annual Meeting of European Association for the Study of the Liver, London, April 9-13, 2014, oral #6.
11. Goossens N, Negro F. Is the genotype 3 of the hepatitis C virus the new villain? Hepatology 2014;59:2403-12.
12. Gastaldi G, Russell A, Golay A, Giacobino JP, Habicht F, Barthassat V, et al. Upregulation of peroxisome proliferator-activated receptor gamma coactivator gene (PGC1A) during weight loss is related to insulin sensitivity but not to energy expenditure. Diabetologia 2007;50:2348-55.
13. Hargreaves M, Kiens B, Richter EA. Effect of increased plasma free fatty acid concentrations on muscle metabolism in exercising men. J Appl Physiol 1991;70:194-201.
14. Tappy L, Schneiter P. Measurement of substrate oxidation in man. Diabetes Metab 1997;23:435-42.
15. Finegood DT, Bergman RN, Vranic M. Estimation of endogenous glucose production during hyperinsulinemic-euglycemic glucose clamps. Comparison of unlabeled and labeled exogenous glucose infusates. Diabetes 1987;36:914-24.
16. Kelley DE, Slasky BS, Janosky J. Skeletal muscle density: effects of obesity and non-insulin-dependent diabetes mellitus. Am J Clin Nutr 1991;54:509-15.
17. Goodpaster BH, Kelley DE, Thaete FL, He J, Ross R. Skeletal muscle attenuation determined by computed tomography is associated with skeletal muscle lipid content. J Appl Physiol 2000;89:104-10.
18. Ma X, Holalkere NS, Kambadakone R A, Mino-Kenudson M, Hahn PF, Sahani DV. Imaging-based quantification of hepatic fat: methods and clinical applications. Radiographics 2009;29:1253-77.

16. ANNEX 1. PRODUCT INFORMATION - 09/07/2015 HARVONI - EMEA/H/C/003850 - IB/0008 - RIBAVIRIN – TABLETS (REBETOL®)

17. ANNEX 2. PROTOCOLS OF D-[6-6-2H2] GLUCOSE SOLUTION PREPARATION (CHUV, LAUSANNE)
